# Supplementary material for: Interspike interval correlations in neuron models with adaptation and correlated noise
Source: PLoS Comput Biol. 2021 Aug 27;17(8):e1009261. doi: 10.1371/journal.pcbi.1009261 (PMC8428727; doi:10.1371/journal.pcbi.1009261)
Supplement: Attachment — Submitted filename: Response to Reviewers.pdf [file pcbi.1009261.s001.pdf]

PLOS Computational Biology Submission
PCOMPBIOL-D-20-00911
Interspike interval correlations in neuron models with adaptation and correlated noise

Reply to the editor's and the reviewers' comments

Dear Prof. Gershman,

thank you very much for your competent handling of our manuscript. We would like to thank both you and the anonymous reviewers for the time and efforts devoted to our work. Below we reply to the reviewers' comments and list all the changes in the manuscript (all changes in the manuscript are highlighted in red). Please find the two requested version of the revised manuscript attached.

Sincerely yours,
Lukas Ramlow and Benjamin Lindner

Reply to First Reviewer

The present manuscript is an analytical modelling study supported by numerical simulations. It describes the second-order statistics for the output spike trains of multi-dimensional integrate-and-fire neuron models in the presence of both a single exponential adaptation current and a correlated noise current in the form of an Ornstein-Uhlenbeck process.

Overall, I think the manuscript reads well and presents the content in an accessible fashion. The authors provide a lot of very insightful intuition about their findings, which, in my eyes, makes the manuscript particularly suitable for publication in PLOS Computational Biology. The authors also start first with special cases and then go on to derive and explain their main theory, which adds to the accessibility of the manuscript.

Most importantly though, a recent experimental finding is explained by new theoretical results in the manuscript, which should make the paper an excellent match for PLOS Computational Biology.

I think the claim that no present model (i.e. integrate-and-fire models without both adaptation and correlated noise input) can generate a correlation structure as presented in Fig. 2(d) is substantiated. Also, the result that a single-exponential adaptation current in a multidimensional integrate-and-fire model (shown in Figs. 5(c) and 6) can induce strong positive serial correlations is very interesting and the interplay of correlated noise and adaptation has never been studied in this setup. Conversely, the authors also show that in the absence of adaptation, a positively correlated noise process can induce negative correlations in a similar model (Fig. 6(b)). As these effects are quite subtle, I would expect that they could be used in a model selection setup to find the best (maybe even unique) minimal model for a given set of noisy spike train observations. As such, the results could prove to be valuable for a wider neuroscience audience.

I mostly have minor comments and suggestions, but before that, I would like to start by suggesting a few more general points to consider.

We very much thank the reviewer for this kind appreciation of our results and our efforts to present them in an accessible manner in this ms.

Major comments

1. *Wouldn't it add to the readability of the manuscript if in Eq. 2, N was set to 1? In the manuscript, the authors only ever look at N=0 or N=1, so maybe the notation could be eased accordingly. If not, I suggest to clearly indicate that $j = 1, .., N$ in Eq. 2b.*

   We do agree with the Reviewer that the notation should be eased as much as possible to improve readability and accessibility for a broader audience. However, we want to emphasize that the possibility to reduce a model with arbitrary dimensions to an one-dimensional phase description, if the phase response curve is accessible, is the essential benefit of the chosen approach. The accessibility of the PRC in turn does not much depend on the number of dimensions but on the complexity of the dynamics, e.g. the involved nonlinearities.

   While we leave the set of equations unchanged to emphasize the generality of the approach, we have added $j = 1, .., N$ on page 3 in Eq. 2b, as suggested by the reviewer (changes are marked in red):

$$\dot{v} = f_0(v, \mathbf{w}) + \mu - a + \eta + \sqrt{2D}\xi_v(t), \tag{2a}$$
$$\dot{w}_j = f_j(v, \mathbf{w}), \qquad j = 1, \dots, N \tag{2b}$$
$$\tau_a \dot{a} = -a + \Delta \sum \delta(t - t_i), \tag{2c}$$
$$\tau_\eta \dot{\eta} = -\eta + \sqrt{2\tau_\eta \sigma^2}\xi_\eta(t). \tag{2d}$$

   Moreover, we have added on page 4 line 100ff a statement regarding the special selection of the considered models which highlights the generality of the method:

   > While our general theory does not impose any restriction on the dimensionality of the chosen IF model we will discuss only a selection of paradigmatic models with one auxiliary variable at most, namely the quadratic IF ($N = 0$), leaky IF ($N = 0$) and generalized IF ($N = 1$) model.

2. *Related to 1., why is the most general case covered by Eq. 2 not treated with an example in the manuscript? In figs. 2-4 and 7, N=0 (in Fig. 2, also D=0), whereas in figs. 5-6, D=0, but N=1. Can the authors please comment on this choice?*

   In most of the examples we have chosen $D = 0$, simply because we think that it adds to the comprehensibility when there is only one noise source at a time. However, it is a good idea to test our theory in the most general case; we have added accordingly a new subsection for the GIF model subject to adaptation and both white and colored noise. Here we also discuss the dependence of the SCC with respect to the ratio of the noise intensities $D/(\tau_\eta \sigma^2)$ and explore the range of validity of our theory (see also point 27 below).

   See new section **Adaptive generalized integrate-and-fire model with both colored and white noise - testing the range of validity** starting on page 11 line 302.

3. *I suggest to reference the paper [47] in list of references contained in the manuscript earlier, and then mention in the Introduction (for example starting on p.3, l.57) that the main new theoretical finding of the manuscript is backed up by experimental findings. The paper [47] seems to be mainly a theoretical study, could the authors additionally cite the original experimental papers on which the study [47] builds?*

   We have modified our statement on page 3 line 62 and cite now the mentioned reference (formerly [47], now [42]) much earlier; we have also added a reference to the original paper (now reference [43]):

   > Our theory predicts qualitatively novel patterns of interval correlations that deviate from a single geometric sequence; such deviations have been recently reported experimentally [42, 43].

4. *It would be good if the authors stated that stationarity of spike trains is assumed to compute the SCC using Eq. 1. [...]*

   As pointed out above, we have added stationarity as a key assumption for our theory on page 5 line 123 as follows:

As pointed out above, we assume that our model in the absence of noise operates in the tonic firing regime with deterministic period T^* and that the spike train in the presence of noise is a stationary stochastic process.

Related to this, could the authors please give additional details on their simulation setup (timestep and algorithms for the numerical integration, sample size to estimate the SCC, sample standard deviations of the SCC across realizations and burn-in period durations). This could be done briefly in the Methods section of the paper.

We added a small subsection **Details on the numerical integration of stochastic equations** starting at page 21 line 518 in the Methods section that contains all requested informations.

Details on the numerical integration of stochastic equations

For the numerical integration of Eq. (2), a stochastic Euler scheme was used with step size $\Delta t = 10^{-5}$. The simulation was terminated after $5 \cdot 10^4$ to 10^5 spikes; for all curves shown the standard error of the estimated mean of ρ_1 was below 0.009.

To reduce transient effects, the initial conditions were chosen to be on the deterministic limit cycle, specifically $v_0 = v_R$, $\mathbf{w}_0 = \mathbf{w}_R$, $a_0 = a^*$, $\eta_0 = 0$. The deterministic peak adaptation value a^* can be calculated from the deterministic period via $a^* = [1-\exp(-T^*/\tau_a)]\Delta/\tau_a$ or, if T^* is analytically inaccessible, directly from integrating the noiseless system. Of course, the specific initial values of the noise variable and adaptation variable in the stochastic system impose a transient (non-stationary) behavior in any measured statistics. However, due to the large number of subsequent ISIs, the transients have little effect on the considered statistics. Indeed, for selected parameter sets, we have verified that the initial values do not matter for the measured SCC.

Minor comments

1. *Concerning the Author Summary (p.1, l.1 ff.), it might be good to mention why SCCs are an important and useful quantity (for example for information transmission and weak signal detection; these considerations are briefly mentioned in the Introduction section and presented in more detail in the Discussion and Conclusion section (p.10)) also with a view on experiments, because, in my opinion, the Author Summary currently is a bit too technical for a general audience of computational neuroscientists.*

   We have change the Authors Summary as follows:

   > The elementary processing units in the central nervous system are neurons that transmit information by short electrical pulses, so called action potentials or spikes. The generation of the action potential is a random process that can be shaped by correlated fluctuations (colored noise) and by adaptation. A consequence of these two ubiquitous features is that the successive time intervals between spikes, the interspike intervals, are not independent but correlated. As these correlations can significantly improve information transmission and weak-signal detection, it is an important task to develop analytical approaches to these statistics for well-established computational models. Here we present a theory of interval correlations for a widely used class of integrate-and-fire models endowed with an adaptation mechanism and subject to correlated fluctuations. We demonstrate which patterns of interval correlations can be expected from the interplay of colored noise, adaptation and intrinsic nonlinear dynamics.

2. *Related to fig. 1: Where is u in Eqs. 2a-d? I understand that Fig. 1 serves to introduce the concept of a phase resetting curve (it becomes clearer after reading the Methods section), but I find the introduction of the new variable u confusing.*

   Interestingly, finding the actual perturbation $u(t)$ is part of the problem. As shown in the Methods section for the most general case considered, the perturbation reads $u_i(\tau) = -\delta a_{i-1} e^{-\tau/\tau_a} + \eta(t_{i-1} + \tau) + \sqrt{2D}\xi_v(t_{i-1} + \tau)$. We are afraid that adding this information to the figure or figure caption would not increase comprehensibility but instead anticipate both the model and the method. Instead, we added a remark in the caption of Figure 1 that emphasizes the fact that finding $u(t)$ is an essential task which is solved in the Methods section:

> Fig 1. Linear response of a neuron models spike timing [...]. Note that separating the perturbation from the deterministic dynamics of the neuron model, i.e. finding $u(t_i + \tau)$ is part of the problem that we address in this paper and solve in detail in the Methods section.

3. *The abbreviation "IF" is introduced multiple times in the manuscript.*

   This has been corrected. In the revised version, the abbreviation 'IF' is introduced once in the introduction on page 2 line 18.

4. *On p.3, l.41, the authors say that spike-frequency adaptation is '...exhibited by almost any type of neuron...'. I don't think this is true in such generality because spike-frequency adaptation seems to be a property mainly of principal (pyramidal) cells.*

   We have relaxed our initial formulation a bit by now stating that *many* neurons are subject to adaptation. We are no Neurophysiologists but from conversations with colleagues and from the literature our impression is that besides pyramidal cells also inhibitory cells such as Martinotti and Vip cells or neurons of the sensory periphery often exhibit spike-frequency adaptation.

   We write now on page 3 line 43:

   > [...] Another mechanism, commonly associated with negative ISI correlations, exhibited by many neurons is spike-frequency adaptation, [...]

5. *p.3, l.47: What is 'adaptation channel noise'? Is it a noisy current caused by the stochastic opening and closing of ion channels mediating an adaptation current? Please explain.*

   The reviewer is absolutely correct in the description of what we mean by adaptation-channel noise. The stochastic opening and closing of the slow adaptation channels provides an adaptation current that we can be split into two parts, correlated fluctuations and a "deterministic" adaptation that is triggered whenever the model fires. We changed the line in question (now page 3 line 49) to make this clearer:

   > In neurons of the sensory periphery adaptation-channel noise, i.e. the stochastic opening and closing of slow ion channels that mediate an adaptation current, may dominate the spiking statistics and provide at the same time adaptation and correlated fluctuations.

   Additionally, we provide a more detailed description on the relation between adaptation-channel noise and our model in the subsection **Leaky integrate-and-fire model with adaptation-channel noise** (see also point 22 below)

6. *p.3, l.57: When mentioning 'novel patterns of interval correlations', it is not clear whether they are theoretical predictions of experimental results (this becomes clearer later in the manuscript). I suggest to point this out already here to make it clear. Related to this, it might also be worth mentioning it in the presentation of the results for the QIF model on p.4, l.98 ff.*

   We now mention explicitly the word 'theory' in the context of novel patterns on page 3 line 60:

   > Our theory predicts qualitatively novel patterns of interval correlations that deviate from a single geometric sequence; such deviations have been recently reported experimentally [42, 43].

   as well as in the context of the QIF model on page 5 line 115:

   > Our theory, that is based on this PRC and detailed in the following, shows excellent agreement with the numerically simulated SCC (theoretical predictions and simulation results are shown in Fig. 2d).

7. *In Eq. 2c, is there a reason why the adaptation jump size is chosen as Δ/τ_a and not Δ, as in previous studies (e.g. reference [38], Schwalger and Lindner 2013, Front Comp Neurosci)?*

   First of all, both kinds of scaling have been used in the literature (for the scaling in the present manuscript see for instance Schwalger et al. PLoS CB 2010). Given a certain parameter set in one scaling, we can of course easily find the corresponding parameter set in the other scaling. In our manuscript, we have opted for this specific scaling because it is the natural choice for the system with

adaptation-channel noise (see Schwalger et al. PLoS CB 2010 or the detailed answer at the very end of this reply letter.)

We emphasized that the chosen scaling is the natural one for a system with adaptation-channel noise in page 4 line 98f:

> In addition, a minor difference of the models is the scaling of the jump amplitude; in [38] the jump amplitude was Δ and not Δ/τ_a but the latter choice is the natural one for systems with adaptation-channel noise.

8. *After their main result (Eq. 3), could the authors comment on how the dynamics of the auxiliary variables w (given by f_j in Eq. 2b) influences the result for the SCC? I assume mainly/exclusively through the phase resetting curve Z. Is this true?*

It is true that the variable w influences the SCC through the PRC. However, also the parameters T^* and via T^* also α , β depend on the features of the subthreshold dynamics, i.e. on the auxiliary variable w. We added a corresponding remark on page 8 line 210 where the GIF model is discussed in detail:

> Having a second variable not only changes the PRC qualitatively but will also affects the deterministic period T^* and, consequently, the parameters α and β.

9. *p.4, l.109 ff.: It would be good to point out that 'weak noise' means small D in Eq. 2a, maybe even already after the presentation of the model equations on p. 3. That is, weak noise mean 'weak white noise' in this paper.*

This is not exactly the case. While it is true that all perturbations that affect the spike timing can eventually be expressed by a perturbation to the voltage variable (see the previous comment on the perturbation $u_i(t)$ or Eq. 30 in the manuscript), this does not only involve the white noise (hence the parameter D) but also η (hence the parameter σ^2). In the revised version, it has hopefully become clearer which parameters have to be small for our theory to work. Specifically we have added a whole subsection devoted to exploring the range of validity of the theory (see **Adaptive generalized integrate-and-fire model with both colored and white noise - testing the range of validity** starting on page 11 line 302):

> Finally, we turn to the most general case and discuss to what extend it can be expected that the SCC of a simulated stochastic spike train is well described by our perturbation theory. We choose a specific parameter set with respect to the deterministic system and the involved time scales and vary the two small parameters of our theory, i.e. the white noise intensity D and variance of the colored noise σ^2. [...]

10. *Is the limit $D = 0$ for $\rho_{k,a}$ in Eq. 3 well-defined? I think it is, because α, β and ν (Eq. 4d) are independent of D and so is $\rho_{k,a}$ (Eq. 5). [...]*

This is correct, the serial-correlation coefficient $\rho_{k,a}$ is independent of D.

However, A and C are dependent on D via $\rho_{1,\eta}$. Does this pose a problem? Or, differently put, what is the SCC in an IF model with adaptation, but without white and colored noise ($D = \sigma = 0$)? [...]

The limit case $D = 0$ and $\sigma = 0$ is well defined if the ratio D/σ^2 remains fixed as $D \to 0$ and $\sigma^2 \to 0$. This can be seen from (the original version of) Eq. 6

$$\rho_{k,\eta} = \frac{\int \mathrm{d}\omega |\tilde{Z}(\omega)|^2 S_{\mathrm{ou}}(\omega) e^{-i\omega T^*}}{\int \mathrm{d}\omega |\tilde{Z}(\omega)|^2 [S_{\mathrm{ou}}(\omega) + 2D]} \beta^{k-1}. \tag{3}$$

in which the power spectrum of the OU process reads $S_{\mathrm{ou}}(\omega) = 2\sigma^2\tau_\eta/(1+\omega^2\tau_\eta^2)$. Divide nominator and denominator by $2\tau_\eta\sigma^2$ to obtain the new Eq. 6 (see below). This term $\rho_{k,\eta}$ is the only term in Eq. 3 that contains the noise intensity D and the variance σ^2. Therefore the limit $D = \tau_\eta\sigma^2 = 0$ is well defined if $D/(\tau_\eta\sigma^2) = \text{const}$.

We have added a remark on page 5 line 124f and changed Eq. 6 so that the dependence of $\rho_{k,\eta}$ on the ratio of the two noise intensities is readily seen:

$$\rho_{k,\eta} = \frac{\int d\omega |\tilde{Z}(\omega)|^2 (1+\omega^2\tau_\eta^2)^{-1} e^{-i\omega T^*}}{\int d\omega |\tilde{Z}(\omega)|^2[(1+\omega^2\tau_\eta^2)^{-1} + D/(\tau_\eta\sigma^2)]}\beta^{k-1}, \quad (6)$$

where $\tilde{Z}(\omega) = \frac{1}{T^*}\int_0^{T^*} d\tau Z(\tau)e^{-i\omega\tau}$. Interestingly, Eq. 6 is the only place where the noise strength parameters D and σ^2 enter and they do so as the ratio of noise intensities $D/(\tau_\eta\sigma^2)$. In this formulation it is simple to see that $\rho_{k,\eta}$ vanishes for $D \gg \tau_\eta\sigma^2$. In the opposite limit of vanishing white noise ($D = 0$), Eq. 6 coincides with the expression derived in [46].

We also added a remark in the Methods section page 17 line 491f:

> Remarkably, Eq. (29) is the only place where the intensity of the white noise and the variance of the colored noise appear in our theory. Note that the limit $D \to 0$, $\sigma^2 \to 0$ is well defined for a fixed ratio of noise intensities, $D/(\tau_\eta\sigma^2)$.

[...] It would be great if the authors commented on it, as this case seems to be treated in Fig. 7(a).

Thank you, our notation in Fig. 7 was misleading because we did not state the value of D for the limiting cases. In the revised version we corrected this in the caption of Fig. 7.

> The limiting cases are shown in orange (no adaptation or white noise, $\Delta = 0, D = 0$) and blue (no colored noise, $\sigma = 0, D = 0.1$).

11. *A (Eq. 4a) and C (Eq. 4c) depend on $\rho_{1,\eta}$ and hence also on properties of the colored noise, whereas the authors say that '...the dependence on the lag k is carried exclusively by the two specific SCCs...'. From the formulas, this is of course true, but maybe it is misleading because it might be misunderstood to mean that the first term in Eq. 3 does not depend on any property of eta and similarly for the second term.*

    We have added a remark that the prefactors occurring in Eq. 3 depend on the properties of the whole system, i.e. adaptation and colored noise (see page 5 line 123 following Eq. 4d).

    > Remarkably, the dependence on the lag k is carried exclusively by the two specific SCCs $\rho_{k,a}$ and $\rho_{k,\eta}$. The prefactors though depend on the properties of the adaptation as well as the noise sources.

12. *p.5, l.121: '...a structure that cannot be generated by a single geometric series...'- Maybe it is good to point out that SCC dependencies on k that are generated by single geometric series need to be either monotonically decaying/ increasing (base > 0, depending on sign of prefactor) or oscillatory and decaying (base < 0) functions of k for completeness and clarity.*

    We have added an explanation that should clarify which patterns can be expected from a single geometric sequence on page 6 line 133ff :

    > Such a sum can exhibit completely different patterns of interval correlations compared to a single geometric series obtained in previous theoretical calculations. We recall that the absolute value of the elements of a geometric sequence $s_k = s_0 r^k$ decay exponentially with the lag k for physically plausible values $|r| < 1$. In addition, the SCC's sign is determined by the prefactor s_0 and the sign between adjacent elements may alternate depending on the sign of the base r. The two possible signs of s_0 and r allow for four distinct patterns of correlations in the case of a single geometric sequence.

13. *p.6, l.135: Could the authors explain the origin of the 'exponential function' that envelops correlation coefficients? Does this envelope come from $\rho_{k,\eta}$?*

    In l. 156 (formerly l. 135) we refer to $\rho_{k,a}$ which is independent of η and can be expressed by a single geometric sequence. What we were trying to explain is that indeed the absolute value $|\rho_{k,a}|$ decays exponentially with k but $\rho_{k,a}$ may have an alternating sign (again depending on the base). Put differently, the geometric sequence can be expressed as follows

$$\rho_{k,a} = \rho_{0,a} r^k = \begin{cases} \rho_{0,a} e^{\ln(r)k}, & \text{if} \quad r > 0, \\ \rho_{0,a} e^{\ln(|r|)k}(-1)^k, & \text{if} \quad r < 0. \end{cases}$$

The second case was referred to as "oscillatory ($[-1]^k$) enveloped by an exponential function" ($e^{\ln(|r|)k}$). The origin of the exponential function is thus due to the fact that $\rho_{k,a}$ can be expressed by a geometric sequence.

14. *p.6, l. 142: The expression given for $Z(T*)$ does not look correct. The bracket should be closed after v_T; what is a^* and why is there a Δ/τ_a appearing? I understand that $T*$ is the deterministic period of the oscillator, which can be obtained by integrating Eq. 2 for $\eta = D = 0$ from v_R to v_T and then solving for $T*$. Please explain.*

    Thank you for the careful inspection. We corrected the expression for $Z(T^*)$. The full term expresses the (inverse) velocity of a deterministic adaptive LIF model at the threshold, i.e. $\dot{v}(T^*) = f(v_T)+\mu-a(v_T)$. The term $a(v_T)$ is the value of the adaptation at the threshold right before it is incremented. Since the deterministic system is periodic there is a fixed peak adaptation value $a(v_R) = a^*$ that is assumed right after the spike and a corresponding value right before the spike $a(v_T)$ that can be obtained by subtracting the increment $a(v_T) = a^* - \Delta/\tau_a$.

    We have added a remark at page 4 line 78ff that explains the variable a^* by means of the deterministic limit cycle:

    > In the absence of any noise we assume that the system approaches a limit cycle (dashed line in Fig.2(b)) with a fixed period T^* and a unique value of the adaptation variable right after a spike, a^*.

    We also corrected the misplaced parentheses in $Z(T^*)$ and added an explanation of $a^* - \Delta/\tau_a$ on page 7 line 163ff:

    > [...] where the prime denotes the derivative with respect to v and $Z(T^*) = [f(v_T)+\mu-a^*+\Delta/\tau_a)]^{-1}$ is the inverse velocity $\dot{v}(T^*)^{-1}$ of the deterministic system at the threshold, see Eq. 15. The term $a^* - \Delta/\tau_a$ corresponds to $a(T^*)$ right *before* the spike (recall that a^* is the deterministic peak adaptation value right *after* the spike)

15. *p.7, l. 149: What is E? It must be related to Eq. 5. Also, on l. 151, an equation reference went away.*

    Thank you for spotting this. We removed the reference and added a small equation on page 7 line 171 that should be more comprehensible:

    > As discussed in the previous section the specific SCC $\rho_{k,a}$ is a geometric sequence with oscillatory or exponential base pattern, distinguished by $\nu < 0$ and $\nu > 0$, respectively. Its prefactor (using $|\alpha\nu| < 1$ and $0 < \alpha < 1$)
    >
    > $$-E(1-\nu) \quad \text{with} \quad E = \frac{\alpha(1-\alpha^2\nu)}{1+\alpha^2-2\alpha^2\nu} > 0$$
    >
    > depends specifically on the PRC and is always negative for positive PRCs.

16. *Related to the explanations on p.7, l. 149 ff.: Can $\rho_{k,\eta}$ (Eq. 6) ever be negative? I would assume not, but on l.152, the authors write: '...and possesses a non-negative prefactor for positive PRCs...'. The PRC enters in Eq. 6 only as the squared magnitude of its Fourier transform, which should always be positive, no matter the sign of Z? Please explain.*

    The SCC $\rho_{k,\eta}$ can be negative. While it is true that $|\tilde{Z}(\omega)|^2 > 0$ and even $S_{ou}(\omega) > 0$ the integral can still be negative due to the term $e^{-i\omega T^*}$. See Fig. 6b where we considered the GIF model without adaptation and showed that the first SCC can be negative for intermediate correlation times. Our reference to Eq. 6 was unfortunate because in this Fourier representation it is not readily seen that a partially negative PRC can lead to negative $\rho_{k,\eta}$. We have changed the corresponding formulation in the text (p. 7, l. 174f) as follows:

    > The second term $\rho_{k,\eta}$ decays exponentially with lag k and possesses a non-negative prefactor because of the positive PRC of the considered model (this becomes evident in Eq. 27).

17. *p.7, l. 156: I think the patterns should be described as 'exponentially increasing or oscillatory', as the SCC at lag 1 is the most negative one (the SCC goes to zero for large lags in the presence of only an adaptation current, like the blue curve in fig. 3(a)). Similarly, this could be changed in the caption of Fig. 3.*

    Although the curve indeed increases with the lag k, we meant something else, namely that the amplitude of the correlation coefficient decays exponentially. In order to make this clear we reformulated on page 7 line 179f as follows:

    > On the contrary, spike-triggered adaptation evokes a negative correlation between adjacent intervals ($k = 1$) followed by an exponential decay in amplitude at higher lags ($k > 1$) that can be either monotonic or oscillatory.

18. *p.7, l.161: Can the authors briefly explain why strong and weak adaptation are parametrized by the sign of nu?*

    We have added an explanation (see below) which is based on the relation $\nu = [f(v_R) + \mu - a^*]Z(0)$. For the convenience of the reviewer we add a short sketch of the derivation (similar to what was done in Schwalger & Lindner 2013, see Appendix 4.1.2):

    Given a one-dimensional IF model with adaptation: First consider the PRC for perturbations in the voltage dynamics that is the solution of $\dot{Z} = -f'(v_0)Z$, where v_0 is the limit-cycle solution of the deterministic system. Second the PRC for perturbations in the adaptation dynamics is determined by $\dot{Z}_a = \frac{1}{\tau_a} Z_a + Z(t)$ which is solved by $Z_a(t) = e^{t/\tau_a}\left(Z_a(0) + \int_0^t dt' Z(t')e^{-t'/\tau_a}\right)$, an expression that has to vanish at the spike time $t = T^*$. Consequently $Z_a(T^*) = 0 = e^{T^*/\tau_a}\left(Z_a(0) + \int_0^{T^*} dt' Z(t')e^{-t'/\tau_a}\right)$. Identifying $\nu = 1 - \frac{a^*}{\tau_a}\int_0^{T^*} dt' Z(t')e^{-t'/\tau_a}$ (see Eq. 4d in the manuscript) in this equation yields $\nu = 1 + \frac{a^*}{\tau_a} Z_a(0)$. Finally, we use the normalization condition stated in Eq. (15) in the manuscript at $t = 0$: $Z(0)\dot{v}_0(0) + Z_a(0)\dot{a}_0(0) = 1$ to find $[f(v_R) + \mu - a^*]Z(0) = \nu$

    We have added a remark on the origin of the naming on page 7f line 185ff

    > We distinguish the two possible cases of strong (a) and weak adaptation (b) in terms of the parameter ν that we can recast into $\nu = (f(v_R) + \mu - a^*)Z(0)$ (derivation similar to [38], Appendix 4.1.2). Strong and weak adaptation are related to the sign of the parameter ν that appears for the noiseless system in the temporal derivative of the voltage at the reset $\dot{v} = \nu/Z(0)$. For weak adaptation ($\nu > 0$) the voltage after reset runs on average towards the threshold. On the contrary, for strong adaptation ($\nu < 0$) the peak adaptation value a^* is so high that the voltage after being reset to v_R drops on average even further towards more hyperpolarized values.

19. *p.8, l.188: The SCC is not positive everywhere in Fig. 5(c), see for example the light green curve. Please explain and correct. [...]*

    Thank you, we have corrected this (p. 9,l. 220):

    > This is seen in Fig. 5(c) where the correlation coefficient for short correlation times are positive.

    I also find it confusing (clearly though, this a novel non-trivial finding) that there is no clear dependence on τ_η in Fig. 5c; for example, the yellow and the green curve differ by only one order of magnitude in τ_η but show completely different behaviors, whereas the two curves with the lowest values for τ_η are encased by the yellow and green curve. I think this is described in the caption of Fig. 5.

    The dependence of the SCC on τ_η in a GIF model with partially negative PRC is indeed non-trivial. For this reason we present Fig. 6 where first SCC is explicitly shown as a function of τ_η. There the reviewer can find exactly the described behavior: Rather weak changes of ρ_1 for small values of τ_η and strong changes within one order of magnitude for $\tau_\eta > T^*$. We have added a sentence on page 9 line 222ff that highlights the connection between Fig. 5c and Fig. 6a as follows.

    > We recall that this case cannot be realized by an LIF model with adaptation and thus represents a novel feature of the GIF model. The involved dependence of ρ_1 on the correlation time τ_η is presented in a different way in Fig. 6(a) and contrasted with a similar case in the absence of adaptation in Fig. 6(b).

20. *p.8, l.194-95: Maybe it would be good to point out that the perturbation u (in terms of Fig. 1) is now negative, so it will advance firing if it acts on the beginning of the next interval where Z is negative.*

We have added a remark on page 9 line 228 that the corresponding inhibitory current is negative.

> A shortened reference interval evokes a positive deviation of the peak adaptation value, $\delta a_i = a_i - a^*$ affecting the next interval. If the corresponding inhibitory (negative) current $[-\delta a_i \exp(-\tau/\tau_a)$, see Methods], acts mainly at the beginning of the next interval where the PRC is negative as well (for instance with $\tau_a \approx T^*/2$ as in Fig. 5(c)), it has a *shortening* effect on the subsequent interval.

21. *p.8, l. 220 and caption of Fig. 7: The authors should describe again which model is considered in detail, for example by noting down the equations or reference earlier models in the manuscript.*

We added a reference to the model at page 10 line 255

> Our model Eq. 2 can also be regarded as an idealized description of [...]

and have changed the caption of Fig. 7 to emphasize how the considered model is related to the initial model (i.e. by setting $\tau_\eta = \tau_a$).

> The SCC of adjacent intervals ρ_1 (black lines and dots) for our model Eq. 2 with identical time constants $\tau_\eta = \tau_a = \tau_c$ [...]

22. *section beginning on p.9, l.219: I suggest to give some more details on the relation of the abstract IF model with a model for a slow stochastic population of adaptation channels, maybe a small sketch is helpful in this regard.*

In the revised manuscript we sketch the relation between stochastic population of adaptation channels and our model (spike-triggered adaptation and colored noise) as follows (page 10, line 255ff):

> Adaptation and colored noise in our model Eq. (2) can also be regarded as an idealized description of the current flowing through a population of stochastically opening and closing ion channels with adaptation-mediating voltage-dependent gating kinetics [33, 34]. A paradigmatic example is the Ca^{2+}-dependent K^+ current [54, 56] but several other candidates for such spike-triggered adaptation currents are known (see [35]). Whatever the type of channel is, the current through a *single* channel is highly stochastic and this remains true also for the summed current through a *finite* population of channels – this is what is commonly referred to as channel noise. As was demonstrated in [33], the total current through a finite population of adaptation channels can be split up into a deterministic part (equivalent to the adaptation dynamics Eq.(2c)) and a stochastic part that corresponds to an Ornstein-Uhlenbeck process (our Eq.(2d)).
>
> In this interpretation of the model, the summed current $a(t) + \eta(t)$ stems from one source, i.e. from the population of adaptation channels and we may regard the sum as an adaptation-channel noise (the white noise may be interpreted as resulting from faster, e.g. Na^+ channels). Crucially, because noise and adaptation have a common origin, the previously independent time constants τ_a and τ_η have to be set equal. We will refer to the common time constant as $\tau_c := \tau_a = \tau_\eta$. Note that consequently we have $\alpha = \beta$, parameters which where defined in Eq.(4d). We emphasize that we will not consider explicit channel models here but refer the interested reader to [33, 34].

23. *related to p.9., l.233 (and also earlier in the manuscript, e.g. p.6, l.134): I find it confusing to call the non-monotonic dependence of the SCC on k 'oscillatory'. Oscillations usually have a time component to them. Maybe it is better to call the behavior 'sign-changing' or simply 'non-monotonic'?*

In the specific example, the lag k serves as a coarse-grained time variable, so the repeating pattern can indeed be regarded as a function of time. In our theory the SCC contains a factor r^k with $r < 0$ in some cases, which, if there would not be any additional damping factor, would correspond to a perfect oscillation. Hence, we respectfully decline the suggestion to use 'non-monotonic' or 'sign-changing' – terms that have a more general meaning from our perspective.

24. *p.9, l.243: 'tau' should be 'τ_c'.*

Thank you, we have added the missing subscript c on page 11 line 288ff

25. *p.9, l.244: What is the 'stochasticity of the adaptation' in the case of weak white noise? Please explain.*

We hope that the added paragraphs under point 22 have made this more comprehensible. Additionally, we added a short parenthetical description on page 11 line 290:

> [...] i.e. the stochasticity of the adaptation (described by the colored noise) wins against the feedback effect of the adaptation

26. *p. 11, equation before l. 336: There should be a reference given for this classical equation.*

We have added a reference to Cox and Lewis (1966) on p 13 line 403

27. *Why do the authors expect that their weak-noise theory also works for stronger noise, as stated in the Discussion and Conclusion section? As far as I can see, it seems that the maximal value considered in the manuscript is D =0.05 in Fig. 7. Please explain.*

We have added a subsection starting on page 11 line 302 that captures the most general case and explores the range of validity of our theory with respect to the two small parameters D and σ^2. As described we find quantitative agreement between simulations and theory up to coefficients of variation of $C_V \approx 0.2$ and qualitative agreement over the whole tested range of $\sigma^2 \in [0, 1]$ and $D \in [0, 1]$. Because of the non-dimensional nature of our model we think that the output variability, quantified in terms of the CV, gives better intuition about the strength of the noise than the absolute values of D and σ^2. The CV is now calculated in the new subsection **Coefficient of variation** in the Methods section.

28. *It would be good to give the numerical values for the deterministic period $T*$ in figs. 5-7 for clarity.*

We have added the deterministic ISI T^* to the caption of figure 4−6. In case of Figure 7 the deterministic period does depend on the time constant τ_c for which reason every data point has an individual T^* so that *the* period can not be stated. We have added some information in the caption of figure 7 about this.

29. *Discussion beginning on p.12, l.364: I don't understand how the two recently proposed techniques to compute the phase of a stochastic oscillator could be used for the computations of the present manuscript, because in my understanding, these two approaches are purely numerical and the techniques in the manuscript require analytical expressions for the PRC. Please explain.*

To start with, we would like to stress that the statements in question are supposed to give an outlook to future work but do not present a worked-out strategy to compute phase responses for stochastic oscillators. The reviewer is right that the original papers putting forward the two notions of phase (Schwabedal & Pikovsky 2013, Thomas & Lindner 2014) used mainly numerical procedures. However, more recently, analytical progress has been made, e.g. on the problem of how to find the isochrones for the mean-first-passage-time phase for a stochastic oscillator as the solution of a PDE (Cao et al. 2020) or even how to calculate them explicitly in case of the asymptotic phase for a multidimensional Ornstein-Uhlenbeck process (Thomas & Lindner 2019). Please, also note that even our present approach is based on some numerical calculations: we have to compute for instance the deterministic limit cycle and the phase-response curve numerically – still we can gain some insights from our theory regarding the general structure of the SCC etc. Similarly, if we have (i) a mapping from multidimensional space of a stochastic oscillator (say an adapting IF neuron with white noise) to one or the other definition of phase and (ii) know the PRC for this phase with respect to an additional perturbation (say an additional colored noise), we would be able to compute non-perturbational expressions for the SCC by similar techniques as developed in our paper (at least, this is what we would hope for). While we have made some progress with (i), we are still lacking any for (ii).

In the revised version we highlight the recent analytical progress as follows (p. 14, l. 436ff):

> ... Indeed, two attempts have been made over the last couple of years to generalize the phase concept for deterministic systems to stochastic oscillators: the phase can be either defined in terms of the mean-first-passage time [86-88] (see in particular the recent analytical approach to the problem in Ref. [89]) or in terms of the asymptotic evolution of the probability density [90] (see specifically the analytically tractable case of a multidimensional Ornstein-Uhlenbeck process addressed in Ref. [91]). It is yet neither clear which of the two phases is more appropriate ...

Comments related to spelling and grammar

We would like to thank the reviewer for the careful inspection. We have corrected the spelling and grammar as suggested.

p.2, caption of Fig. 1: 'integrated' instead of 'integrate'.
p.2, l.30: there should be no comma after 'although'.
p.2, l.33: Missing 'e' in 'therefor'.
p.3, l.45: It should be 'by far' and not 'far'.
p.3, l.51: 'correlation-inducing' instead of 'correlation inducing'
p.6., caption of Fig. 3: 'case shown' should be 'case is shown'.
p.6, l.145: 'brings' instead of 'bring'
p.6, caption of Fig. 4: 'SCCs' should be 'SCC'.
p.7, l.170: 'like' should be 'similar'.
p.8, caption of Fig. 6: 'effected' should be 'affected'.
p.8, l.207: comma missing after 'T'*
p.9, caption of Fig. 7: 'to' should be 'so'.
p.10, l. 285: 'in order predict' should be 'in order to predict'.
p.17, l.436: typos: 'proceeding' should be 'preceeding' and around 'device'.
p.18, l.441: 'ms' should be 'manuscript'.
There is one comma and a full stop missing at the end of the captions for figures 5 and 6.

Reply to Second Reviewer

Ramlow and Lindner's work calculates serial correlations between adjacent spikes in a multidimensional integrate-and-fire neuronal model with temporal input correlation, and the spike-triggered spike-frequency adaptation. Their method is based on the standard calculation of the Phase-Response Curve (PRC) for the neuron models in a noise-less setting and then averaging it over a weak noise realization. The method has severe limitations; it requires a substantial mean input far beyond the spiking threshold and feeble noise. Also, adaptation and temporal noise correlation should have both rather long time-constants. Using this method, they report that in both cases of a temporally correlated input and the spike-frequency adaptation, one cannot approximate the inter-spike-interval serial correlation using a geometric series. The other reported technical result is that the inter-spike serial correlations can be negative in a neuronal resonator model due to PRC's shape, while neurons receive a colored noise input in some parameter range.

We disagree with this summary of our work in several respects:

1. **Our analytical approach as a standard ansatz**
While our approach relies on a perturbation ansatz as used in many other fields, it is still not as straightforward as suggested by the reviewer. The above summarizing statement omits that we have to establish stochastic maps for the deviations of adaptation variable and ISIs and only after a number of nontrivial manipulations we arrive at a stage at which we can average over the noise realizations.

2. **Limitations of the results: substantial mean input**
As we openly discuss in our ms, all our analytical results are indeed restricted to neurons in the mean-driven regime. This is not as useless as claimed by the reviewer because many of the important examples for non-vanishing serial interspike interval correlations stem from the sensory periphery (e.g. P-units in weakly electric fish, electroreceptors in paddle fish, auditory receptor neurons in grasshoppers), where neurons often operate in the mean-driven firing regime. Our theory thus applies to all these interesting and physiologically relevant cases. In addition, apart from this assumption, our model is rather general (i.e. not subject to the limitations of many other studies) in other respects: the nature of the subthreshold spike generator, the correlation time of the noise, and the inclusion of an adaptation current with arbitrary time constant.

   Excitable (fluctuation-driven) neurons as encountered predominantly in higher brain regions cannot be readily approached with our PRC-based theory. However, as pointed out in the discussion section, we hope that in the near future concepts like the PRC can also be used in a stochastic framework and that some of the methods developed here will be then also applicable to excitable neurons.

3. **Limitations of the results: feeble noise**
It is correct that for some of the plots we have used rather small values of the noise intensity or variance. In order to give some intuition about the range of output variability for which our theory is still valid, we have used the most general setup of the model (a GIF model with adaptation and both white and colored fluctuations) and separately varied the strength of the two noise sources (new fig. 8 in new subsection **Adaptive generalized integrate-and-fire model with both colored and white noise - testing the range of validity**). In terms of the output variability it becomes clear in this plot that the theory is robust enough such that it predicts the SCC quantitatively up to CVs of about 0.2 (sometimes a bit lower, sometimes a bit higher) and that it qualitatively matches typical correlation patterns (i.e. SCC as a function of the lag) for an even larger range of CVs (up to CV=0.7). CVs of sensory neurons that show strong negative correlations are in this range (see e.g. Grewe et al. PNAS 2017, fig.1E). Summarizing, there is no need to consider only feeble noise – as illustrated in the new fig. 8 in the revised manuscript, our theory has a broad range of applicability. We have added a corresponding statement to the abstract.

4. **Limitations of the results: long time constants for adaptation and correlated noise**
This is a puzzling remark - it is a strength of our method and it was demonstrated several times in our manuscript that our theory holds true for arbitrary time constants of noise and adaptation. This is illustrated by a number of plots that show the excellent agreement of the theoretical SCC with numerical simulations as a function of τ_η or both τ_a and τ_η as in the case of channel noise. In these comparing figures the time constants start at values much smaller than the unperturbed ISI, i.e. here the time constants are definitely short.

5. **Only key results: correlations beyond a single geometric sequence and negative SCC for resonator model driven by colored noise**
While the two mentioned findings are important results of our study, we think that the referee omitted important further results:

   - Our paper constitutes the first analytical study of a neuron with two sources of ISI correlations and thus provides first intuition for this experimentally relevant case (most neurons are subject to both adaptation and correlated fluctuations).
   - We not only demonstrate that the SCC cannot be captured by a single geometric sequence but we derive explicitly that it attains the form of a sum of two geometric sequences.
   - We present the first calculation of the SCC for the case of stochastic adaptation-channel noise that includes both the correlated fluctuations as well as the adaptation effect of the channels (Schwalger et al. 2010 captured only one of these aspect at a time).
   - We show how to control the sign of ISI correlations by changing the relative strengths of colored and white noise sources.

   Some of these aspects are hopefully more clear in the revised version of the ms. We also reiterate (see above) that we now give some insight into the range of parameters (in particular noise strengths) for which our theory holds true.

Detailed criticism

The present method is a rather standard approach in physics and computational/theoretical neuroscience (see, for example, classical Ermentrout (1996)). Also, I believe the manuscript lacks new results, and its limiting assumption for the calculation is major. Additionally, I do not see how these limited technicalities can be relevant to the border readership of PLoS Computational Biology.

As already outlined above, we do not think that our approach is so common and we think that the reviewer misunderstood what we did. First of all, he/she got one of the assumptions of our theory wrong: there is no need to have a slow noise and/or a slow adaptation! Secondly, the reviewers description seems to assume that we just write up the perturbation in the ISI due to the external noise and then have to average the product of distinct ISI deviations over the noise – this is true if we exclusively deal with colored noise as in subsection **Correlation coefficient for IF models without adaptation** but certainly (and interestingly)

not in the general case with both colored noise and adaptation. Writing up the deviations in time intervals and adaptation variable caused by the external fluctuations, we end up with coupled stochastic maps of these deviations that can be, with a number of nontrivial manipulations, brought to a form for the covariances of the lagged ISIs that can be averaged over the noise ensemble. The full calculation for the system with colored noise and adaptation fills approximately six pages (see methods section) and appears thus not as such a standard task as claimed by the reviewer. If he/she knows a shorter way, we would be eager to learn about it.

The PRC's application in this setting strongly requires that the noisy input does not alter the spiking frequency of the neural models. This assumption is majorly incorrect in a wide range of physiological parameters where the serial correlations can be relevant (for example, see spiking CV in Chacron et al. 2003). One can see the parameters in Fig.3: The mean input μ is set to be 5 or 20 while the temporal variance σ^2 of colored noise is set to 0.02, and for white noise, they use 0.001. Similarly, one can see these enormous differences in Fig.4-7. It is unclear to what extent one can expect the theory to fit the simulations.

Although Maurice Chacron is a co-author to one of us, we have trouble identifying the paper that the reviewer refers to (Chacron et al. 2003). As pointed out above, CVs of sensory receptor neurons such as P-unit in weakly electric fish (a system that Chacron et al. worked on a lot in the early 2000's) are between 0.1-0.8. In our new Fig. 8 in the revised manuscript we show that our theory applies quantitatively up to CVs of 0.3 and makes qualitative predictions that hold true for even higher CVs. We would thus claim that our theory is applicable to physiologically relevant cases.

In higher dimensional models, similar to the resonator example described in Eqs 9a and 9b, the PRC reflects the system's phase along the limit cycle. In these models, one expects that the fluctuation along the orthogonal dimension also contributes in the same order to the serial correlations. However, those contributions are not present in the current analysis on page 8/23.

The reviewer presents here an entirely new argument indicating that he/she thinks that the perturbation calculation is not as standard as claimed before (see above). For a start, as anecdotal evidence for the quality of our theory we mention the excellent agreement of our analytical expression with numerical simulations for a large number of special cases.
More importantly, there are analytical arguments. According to a recent reference on the topic (Wilson PRE, 2019), the phase-response curve can be generalized to a phase-amplitude-response curve by considering perturbations along orthogonal dimensions (amplitude) as follows. First, consider the phase dynamics perturbed by a weak signal $\epsilon u(t)$

$$\dot{\theta} = \frac{\partial \theta}{\partial \mathbf{x}} \frac{d\mathbf{x}}{dt} = \omega + \frac{\partial \theta}{\partial \mathbf{x}} \epsilon u(t).$$

The derivative $\partial\theta/\partial\mathbf{x}$ is evaluated with respect to a small perturbation to the limit cycle $\mathbf{x}^\gamma(\theta) + \epsilon\Delta\mathbf{x} + O(\epsilon^2)$; here $\mathbf{x}^\gamma(\theta)$ the limit-cycle solution and $\epsilon\Delta\mathbf{x}$ is the linear response deviation due to the weak perturbation $\epsilon u(t)$. This can be expanded as follows:

$$\left.\frac{\partial \theta}{\partial \mathbf{x}}\right|_{\mathbf{x}^\gamma(\theta)+\epsilon\Delta\mathbf{x}+O(\epsilon^2)} = \left.\frac{\partial \theta}{\partial \mathbf{x}}\right|_{\mathbf{x}^\gamma(\theta)} + \left.\frac{\partial^2 \theta}{\partial \mathbf{x}^2}\right|_{\mathbf{x}^\gamma(\theta)} \epsilon\Delta\mathbf{x} + O(|\epsilon\Delta\mathbf{x}|^2)$$

the first term is the phase response curve $(O(\epsilon^0))$ the term that contains second derivative is of order ϵ (eventually ϵ^2 if multiplied by $\epsilon u(t)$) and can be expressed by means of the isostables ψ_j

$$\dot{\theta} = \omega + \epsilon \mathbf{Z}(\theta)\mathbf{u}(t) + \epsilon^2 \sum_{k=1}^{n-1} [\mathbf{B}^k(\theta)\psi_k]\mathbf{u}(t),$$

$$\dot{\psi}_j = \kappa_j \psi_j + \epsilon \mathbf{I}_j(\theta)\mathbf{u}(t) + \epsilon^2 \sum_{k=1}^{n-1} [\mathbf{C}_j^k(\theta)\psi_k]\mathbf{u}(t).$$

Because of the assumed stability of the limit cycle we now that $\kappa_j < 0, \forall j$ and consequently ψ_j vanishes in the absence of the perturbation. Note that we have changed the notation of Wilson a little to emphasize the

occurring terms dependence on ϵ - however, Wilson writes on page 3 *"One strategy for computation of* $\mathbf{B}^k(\theta)$ *and* $\mathbf{C}_j^k(\theta)$ *[which characterize the* $O(\epsilon^2)$ *terms (...)] is to (...)".*

To summarize fluctuations along the orthogonal dimension, i.e. amplitude, affect the interspike interval only in higher order ($O(\epsilon^2)$).

We have added a remark on this that points the readers attention towards phase-amplitude-response curves on page 14 line 427:

> Of course, these cases are of particular importance for the stochastic dynamics of cortical cells and they can be addressed in the Fokker-Planck framework as has been demonstrated for important spike-train statistics in full generality in [44]. Cases in which strong noise impinges on a mean-driven neuron may be also addressed by taking into account amplitude dynamics [87, 88] and higher order phase-response functions.

As a reader of PLoS CB, I'd consider this submission a technicality, and it lacks sufficient new results. Therefore, I advise a specialized journal could be more suitable for this work.

Further changes

1. We have changed the first and second sentence of the Abstract. As follows:

   > The generation of neural action potentials (spikes) is random but nevertheless may result in a rich statistical structure of the spike sequence. In particular, contrary to the popular renewal assumption of theoreticians, the intervals between adjacent spikes are often correlated.

2. After consulting a native speaker, we have changed the term 'geometric series' to 'geometric sequence'.
3. In Eq. 7 the partial derivative ∂_v was replace by a prime.
4. In Fig. 8 we compare the theoretically predicted and simulated SCC ρ_1 as well as coefficient of variation (CV). Because the CV was not calculated in the original manuscript we have added a new subsection **Coefficient of variation** in the **Methods** section where we derive an expression for the coefficient of variation for the full system.

Appendix

Regarding point 7 of the first Reviewer:

In more detail we follow reference [33] to derived the appropriate scaling of our Model Eq. (2c-d) with respect to τ_c. To this end, consider the stochastic opening and closing of a population of $N = N_{\text{open}} + N_{\text{closed}}$ ion channels

$$N_{\text{open}} \xrightarrow{\alpha_w N_{\text{closed}}} N_{\text{open}} + 1, \tag{7}$$

$$N_{\text{open}} \xrightarrow{\beta_w N_{\text{open}}} N_{\text{open}} - 1. \tag{8}$$

N_{open} is the number of open channels that corresponds to an adaptation current that is eventually approximated by $a(t) + \eta(t)$. The rates are conveniently chosen to be $\alpha_w(V) = w_\infty(V)/\tau_w$ and $\beta_w(V) = [1 - w_\infty(V)]/\tau_w$. The corresponding master equation for the probability to find a channel in the open state w reads

$$\dot{w} = \alpha_w(V)(1 - w) - \beta_w(V)w, \tag{9}$$

or, if expressed in the common form of an voltage-depended steady-state open probability $w_\infty(V)$ and time constant τ_w,

$$\tau_w \dot{w} = -w + w_\infty(V). \tag{10}$$

We reduce $w_\infty(V)$ further so that the channels may only open when the membrane potential is sufficiently depolarized, i.e. during an action potential. This allows to relate the steady-state open probability $w_\infty(V)$

to the spike times t_i: $w_\infty(t) = \sum_i \theta(t-t_i)\theta(t_i+\tau_{\rm AP}-t)$. Here $\theta(t)$ is the Heaviside function, t_i are the spike times and $\tau_{\rm AP}$ is the duration of the action potential.

A corresponding Langevin equation can be derived by means of the diffusion approximation if there is a time window during which $N_{\rm open}$ does not change considerably yet the number of transitions is large enough to be treated as a Gaussian random number. In that case the fraction of open channels can be approximated by a Langevin equation as follows:

$$\tau_W \dot{W} = -W + W_\infty(t) + \sqrt{2\tau_W \sigma^2/N}\xi(t). \tag{11}$$

where $\xi(t)$ is Gaussian white noise. We separate the fraction of open channel W into an deterministic part $a(t)$ and a stochastic part $\eta(t)$ by choosing $W = a + \eta$ and obtain

$$\tau_W \dot{a} = -a + a_\infty(t), \tag{12}$$

$$\tau_W \dot{\eta} = -\eta + \sqrt{2\tau_w \sigma^2/N}\xi(t). \tag{13}$$

Finally, in the manuscript we considered an IF model where the spike is not explicitly modeled, thus $\tau_{\rm AP} = 0$, yet the "deterministic" fraction of channels (described by the variable a) that open during a spike should be preserved, i.e. $\int_{t_i}^{t_i+\tau_{\rm AP}} w_\infty(t)dt = \tau_{\rm AP} = \Delta$, where we used the approximation $w_\infty(t) = \sum_i \theta(t-t_i)\theta(t_i + \tau_{\rm AP} - t)$. Put differently, in an IF model where the spike is only a point in time the adaptation variable should be incremented by a fixed value whenever a spike is fired and the scaling with τ_c should be chosen as follows:

$$\tau_W \dot{a} = -a + \Delta \sum \delta(t-t_i), \tag{14}$$

$$\tau_W \dot{\eta} = -\eta + \sqrt{2\tau_W \sigma^2/N}\xi(t). \tag{15}$$

Because these arguments have been presented in detail in reference [33] we think that we do not need to repeat them in our manuscript.
