# Supplementary material for: Interspike interval correlations in neuron models with adaptation and correlated noise
Source: PLoS Comput Biol. 2021 Aug 27;17(8):e1009261. doi: 10.1371/journal.pcbi.1009261 (PMC8428727; doi:10.1371/journal.pcbi.1009261)
Supplement: Attachment — Submitted filename: Response to Reviewers.pdf [file pcbi.1009261.s002.pdf]

PLOS Computational Biology Submission
PCOMPBIOL-D-20-00911

Interspike interval correlations in neuron models with adaptation and correlated noise

Reply to the editors and the second reviewers comments

Dear Prof. Gershman, Berlin, January 5, 2020

thank you very much for the opportunity to resubmit our manuscript.

As we have argued in our appeal letter and repeat below for consideration by alternative reviewers, we disagree with the three major claims made by the second referee with respect to our paper. Consequently, in this second resubmission we have made only one minor change: we now cite the paper by Ermentrout et al. J. Comput. Neurosci.(2011) because one of our analytical expressions (a by-product rather than one of the central results), the variance of the interspike interval, agrees in a particularly simple limit case (only white noise, no colored noise, no adaptation) with a formula by these authors.

We hope these changes together with an independent evaluation by a third reviewer will permit a positive decision on our manuscript.

Sincerely yours,
Lukas Ramlow and Benjamin Lindner

Detailed response to the claims of Reviewer #2

Failure of this Reviewer to grasp the scope and content of our paper

Before we come to the three arguments the Reviewer put forward we want to emphasize that the Reviewer seems to continue to misunderstand the scope of our theory. He/She claims that we consider a "*leaky integrate-and-fire (LIF) model with an additional slow variable (colored noise and adaptation)*". This is wrong for two reason. We are not bound to consider a specific model as long as the PRC is analytically accessible. We demonstrated this for two canonical models, namely the LIF and generalized IF model. Moreover, we do not have to impose any restrictions on the spike-triggered adaptation or time-constant of the colored noise - it can be slow, fast or possess a time scale that is similar to the neural spike generator. This is an important point because the slowness of adaptation and/or noise was a crucial assumption in previous theories. It is a strength of our theory that we are not limited to the specific time scales of the adaptation and colored noise, respectively.

Additionally, the Reviewer seems to take the result of a didactical derivation (the correlation coefficient for a noise-driven IF neuron without adaptation, Eq. (24)) for our main result. As we point out in the manuscript, the special result Eq. (24) agrees with published and numerically confirmed results from ref. Schwalger et al. (J. Comp. Neuroscie. 2015) [the Reviewer's criticism should then also apply to this reference, by the way]. He/She misses that our main result is given in Eqs. (45-47), which is the correlation coefficient for an adapting neuron driven by colored noise.

Argument I: Lack of novelty of our approach - Citations to crucial papers with related results are missing

The Reviewer claims that we have missed to cite papers which are essential contributions on which our approach and our results build. Related to this, he/she claims that our approach is not sufficiently novel to merit publication in the Journal. Specifically, the Reviewer writes

1- Novelty of the approach

In the first review comment, I indicated that the developed theory (if it is correct) is based on an approach that is standard and does not go beyond previous works methodologically. The authors disagreed. Note that I do not mean that there are no open theoretical questions in the methodology, but in contrast, one can develop standard methods to tackle unsolved problems. However, the authors are silent about those critical issues in using phase-response formalism to compute higher-order spiking statistics. Those issues make the current theory mathematically wrong (See point 2 in this report). Here, I include a few papers using the same methodology to show the approach's historical scope to account for spiking statistics using phase-reduction methods.

Ermentrout et al. (J Comput Neurosci 31, 2011) calculate ISIs distribution's mean and variance for a general multi-dimensional oscillator dynamics with additive Gaussian noise. Their results presented on pages 20 and 21 under the Coefficient of variation are specific examples of this paper. This paper is not cited.

Ota et al. (PLoS One, 2012) calculated higher-order statistics of neural oscillators. Importantly, this work shows spike covariance (eq. 11), a related quantity to the authors' primary results in eq. 24, which must be evaluated up to higher orders in the perturbation expansion. These additional terms are missing in the authors' calculation (see point 2 in this report). This paper is also, unfortunately, not cited.

Another very relevant work, which is missing in the references, is the paper by Teramae et al. (PRL102, 2009). In this paper, they have studied a general oscillator driven by colored noise. This is indeed similar to the model in eq. 2 of this submission. The result here indicates that a noisy oscillator phase's dynamics do have an order-one term orthogonal to the limit-cycle as well, see $k(\phi)$ *in equation 12 therein, and therefore cannot be neglected. This term is missing in the authors' calculation of the serial ISI correlations without any reasoning.*

They are plenty of more similar examples in the literature. However, I end this list with a paper by van Vreeswijk (PRL 84, 2000). In that paper, van Vreeswijk shows in a general setting, as a result of a perturbation, the timing of n-th spikes has a higher-order contribution from the corresponding Floquet

exponent (eq. 6-9). These contributions are essential for calculating the interspike-intervals serial correlations. Moreover, this work includes the specific case of spike-triggered-adaptation, and it has also derived the additional contributions in perturbation series (see eq. 18 and 19 therein). The work here is highly relevant for authors, but it is overlooked in Ramlow and Lindner's revision.

Now, there are a lot of papers that deal with stochastic oscillators, both experimentally and theoretically and still many theory papers among those apply perturbation methods to calculate some statistics of interest. Obviously, we cannot cite all of these papers - this would be neither interesting nor useful for the reader. We think it is appropriate and also required to cite results put forward on the problem of interspike interval correlations and on neural oscillators driven by colored noise and adaptation. This is what we did and we cited already quite a number of papers.

For the papers cited by the Reviewer one can easily verify that

- none of them deals with the central subject of our study, namely, interspike interval (ISI) correlations, their pattern with respect to the lag, their dependence on noise and adaptation parameters etc. In fact, none of the models in the cited papers would generate ISI sequences with ISI correlations (they are all renewal models);
- adaptation is only considered in a noiseless setting by Van Vreeswijk but in none of the other papers;
- none of the papers considers colored noise: The paper by Ermentrout et al., Teramae et al. and Ota et al. deal with phase oscillators driven by *white* noise (Teramae starts with a colored noise setting but is solely interested in the white-noise limit of this model), whereas the model studied by Van Vreeswijk does not consider any noise at all.

To be very careful here: only one of our results coincides in a very special case with a formerly derived statistics by Ermentrout et al. (unnumbered Eq. after their Eq. (10)): the ISI variance of a noisy oscillator, Eq. (27) in a setting *without* adaptation and *without* colored noise ($\sigma = 0$). So neither this special case (a renewal case *without* ISI correlations) nor the specific statistics (the ISI variance and not the ISI correlation coefficient) are important for our study. However, we would like to emphasize that our result in this case agrees with that of Ermentrout et al. (another check for the correctness of our approach that the Reviewer did not bother to carry out). Hence, we are happy to cite the paper by Ermentrout as a consistency check for our result of the ISI variance in a special case (p. 17, l. 492 of the revised manuscript):

> We note that the variance of the interspike interval in the case of purely white noise (Eq. (27) with $k = 0$ and $\sigma^2 = 0$) reduces to $\langle \delta T_i^2 \rangle = 2D \int_0^{T^*} d\tau Z(\tau)^2$, which agrees with an expression derived by Ermentrout et al. [101] (see the unnumbered equation below their Eq. 10).

Hence, neither with respect to the model ingredients nor with respect to the central statistics of interest are the cited papers relevant to our study and it is puzzling how this Reviewer could come to the conclusion that they should be relevant for us. We must say that the Reviewer's move to first complain about missing references without stating any (in his/her first review) and then to give specific but irrelevant references in his/her second review is especially unfair to us - would he/she have given these references in his/her first review, we could have easily refuted the corresponding claims of strong relevance for our work and possibly convinced the editor that this Reviewer lacks proper competence to judge our work.

To further support our point of view, we briefly outline the content of the papers and present them in even more details in the appendix.

1. Ermentrout et al. (J. Comput. Neurosci. 31, 2011) are concerned with an experimentally motivated setup, i.e. with the variability of the (deterministic) PRC in a setting with uncorrelated (white) noise and no adaptation. They calculate the mean and variance of the PRC and obtain as a side result the variance of the ISI for a renewal case as outlined above. We have no idea why the Reviewer claims that "*their results presented on pages 20 and 21 under the Coefficient of variation are specific examples of this paper*". It is the other way around: our way more general result Eq. (53-55) agrees in a very special case (no adaptation, no correlated but only white noise) with their simple result (the unnumbered eq. after their Eq. (10)). With respect to the criticism of our weak-noise assumption, we note that Ermentrout et al. in the calculation of their statistics of interest (the variance of the PRC) use the same perturbation approach and take into account only the *leading* order of the noise amplitude.

2. Ota et al. (PLoS One, 2012) calculate the spike-triggered covariance (STC) in the most generic setup, i.e. a system solely perturbed by white noise with small amplitude ε. The statistics of interest is here fundamentally different: an average of the driving signal conditioned on the spikes of the system. This cannot be easily connected to the statistics of the interspike intervals - this is neither done in the paper by Ota et al. nor by the Reviewer in his/her report. Because the noise is uncorrelated (and thus will generate only a renewal spike train of the driven neuron model) it affects the STC only in higher-order ($O(\varepsilon^4)$). We incidentally note that the way Ota et al. take into account higher orders in the signal amplitude casts some doubts on the validity of their approach: higher-order corrections should be taken into account by higher-order response functions and not by products of the linear-response function. Again we are not sure why the Reviewer brings this study into play that is concerned with a very different statistics.

3. Teramae et al. (Phys. Rev. Lett. 2009) deal at the first glance with a problem related to what we study in our paper: a neural oscillator driven by a correlated Ornstein-Uhlenbeck noise. They derive an augmented phase description taking into account perturbations to the phase as well as amplitude dynamics. However, the authors are exclusively interested in a white-noise limit case for the driving fluctuations, in which they also let the time scale of the amplitude fluctuations amplitude shrink to zero. Hence, they solely consider a renewal case without colored noise and without adaptation and, consequently, no ISI correlations are studied in this paper (they would be simply zero in this case). In this limit their result Eq. (11) and (12) demonstrates that amplitude dynamics contributes in second order of the noise amplitude to the phase. This is exactly what we argued before; perturbations to the amplitude of order-one contribute to the phase in order-two (see also next point).

4. Van Vreeswijk (Phys. Rev. Lett. 2004) considers a problem of mean-field theory: a network of noiseless limit-cycle oscillators coupled *strongly* in an all-to-all connection topology. Van Vreeswijk is interested to identify conditions under which an asynchronous state of the network is stable and to this end performs a perturbation calculation to study this state's stability. As one example, the author considers an integrate-and-fire neuron endowed with an adaptation dynamics and demonstrates that in the limit of strong coupling among the units, the network can become unstable. As we are not concerned with a network, we have noisy instead of deterministic neurons, and we deal with the limit of weak input instead of strong input (as Van Vreeswijk does), it remains unclear how the results by Van Vreeswijk are related to our results in any way.

In conclusion, the Reviewer's claim that we have missed important references and that these references would already cover essential parts of our method is not justified.

Argument II: Mathematical derivation is flawed by ignoring certain terms in the calculation that may contribute (in leading order) to the correlation coefficient

Secondly, the Reviewer cites a number of paper to argue why he/she thinks that our theory is mathematically incorrect. He/she claims that we should consider higher-order terms for higher-order statistics. This may sound convincing but is mathematically wrong. Specifically, the Reviewer writes:

> *2- Mathematical correctness*
>
> *In the first review report, I asked why the contribution of the fluctuation along the orthogonal dimensions to the limit cycle is absent in the calculation. The authors responded by citing a Wilson (PRE, 2019) paper to justify why they neglect higher-order terms. The result by Wilson (PRE, 2019) is an asymptotic result for the phase-amplitude reduction (they are more insightful and accessible papers on this topic; for instance, please look at Wedgwood et al. (J Math. Neurosci 3, 2013), Castejon et al. (J Math Neurosci 3, 2013) and Perez-Cervera et al. (Chaos 30, 2020)). Wilson (PRE, 2019) projects dimensions orthogonal to the limit-cycle into isostables structure to drive the reduction. By definition, the isostables define as the space that perturbations go back asymptotically to the same limit-cycle. This is irrelevant for the closeby ISIs as the authors study it here. Therefore, this only argument of the authors is not convening, and it is unclear why simulations and mathematical derivations can fit at all in some range.*
>
> *Moreover, as it is shortly mentioned above in works by van Vreeswijk (PRL84, 2000), Teramae et al. (PRL102, 2009), and specially Ota et al. (PLoS One, 2012) they all show that the higher-order terms have to contribute to higher-order statistics of ISIs. This is an exciting and open problem in the realm of this standard approach, and unfortunately, it is overlooked.*

One can understand this intuitively. Let's consider a noiseless system with a periodic solution, and it happily moves along its trajectory. A small stochastic perturbation shifts its time to the first spike as it changes the trajectory shape temporarily. Therefore, it also contributes to the second spike (especially if it has a slower variable in its Cartesian description). The trajectory does not automatically reset to its equilibrium starting point after just one cycle. The shape of the phase-response function only goes back to its steady-state with some non-trivial timescale. This is very nicely explained by Teramae et al. (PRL102, 2009) for a general system with a limit-cycle driven by colored noise (similar to the model that Ramlow and Lindner consider in this manuscript). Teramae et al. (PRL102, 2009) clearly state that the orthogonal direction's fluctuation to the limit cycle remains order-one (see the text above the eq. 3 therein). This is important if one needs to calculate the correlation of two back-to-back interspike-intervals.

Let me rephrase to make sure that this comment arrives at the authors in the most collegial way. In eq. 19 in the submission, we have only the perturbation along the limit-cycle because we define $u_i(t) = \epsilon\delta(\tau - \tau_0)$ in that sense. However, in eq. 2 (Cartesian description), the noise also has components along the orthogonal dimension to the limit-cycle as it is written in eq. 2 in Teramae et al. (PRL102, 2009). Therefore, when the limit-cycle is stable, one must introduce an amplitude-relaxation time for it, which remains order-one after the perturbation. Missing the correct transformation makes the perturbation series truncated wrongly. Thus, by looking at the calculation in van Vreeswijk (PRL84, 2000), Ota et al. (PLoS One, 2012), and Teramae et al. (PRL102, 2009), I am convinced that the theory developed here is mathematically wrong (in the sense of incomplete perturbation expansion).

We stress again that our theory is based on the **linear**-response function of a phase model, namely the phase-response curve, a description that holds true only in leading order of the perturbation amplitude σ. Therefore, perturbations with amplitude σ, to the phase and in consequence to the interspike-interval can meaningfully be describe up to linear order, i.e. $O(\sigma)$. This also implies that averages of products of intervals depend in leading order on σ^2 . Any extension into the realm of higher-order terms is arbitrary as long as it does not stem from a systematic nonlinear-response function of order two. We are surprised to learn that the Reviewer seems to argue that a linear-response theory in the context of phase-reduced models cannot be performed, especially since this approach has been so successful in neuroscience. The paper that are indeed concerned with a systematic derivation of second-order responses (that is Teramae et al. and Wedgwood et al., Castejon et al., Perez-Cervera et al.) all report that the resulting additional terms to the phase dynamics are of order two with respect to the perturbation amplitude (hence, they would contribute in even higher order than σ^2 to the interval correlation coefficient). Hence, our theory is *correct in leading order of the perturbation amplitude* and it is thus no surprise that "*simulations and mathematical derivations can fit at all*" as the Reviewer puts it.

We have argued in detail with the Reviewer's claim of his/her first report that the amplitude dynamics may contribute in leading order to ISI correlations but unfortunately, he/she did not respond to the detailed calculation (based on Wilson's recent PRE paper) that we gave in our reply. Instead he/she argues qualitatively that Wilson's focus on iso-stables would not apply if we wanted to calculate correlations between adjacent ISIs.

In addition, we note that the Reviewer's line of argument is confused with respect to the loose usage of the word *amplitude*: there is a perturbation amplitude (the standard deviation of the colored noise, σ, for our system), there is the amplitude from the phase-amplitude relation (where amplitude stands for the deviation of the multidimensional oscillator from the deterministic limit cycle), and there is the amplitude of time-scale separation (of the colored noise from the limit-cycle dynamics) used in the paper by Teramae as $\epsilon = \sqrt{\tau_\eta}$ (where τ_η is the correlation time of the Ornstein-Uhlenbeck noise that is considered by Teramae et al. in the limit of white noise, $\tau_\eta \to 0$). The first two amplitudes will be related (this was the subject of our first rebuttal and the argument that the amplitude dynamics does not contribute in leading order to the ISI correlations), while the third 'amplitude', the square root of a time constant, is a small parameter only by the choice of the authors (Teramae et al.) to take a white-noise limit that is of no concern for our paper. The Reviewer mixes merrily all these different amplitudes in his/her arguments (specifically in the concluding sentence of the last paragraph quoted above) and claims that we do not take them properly into account, comparing thus (or better, setting equal) apples and oranges.

It is also our impression that regarding the calculation, the Reviewer never got beyond our didactical example of a purely colored-noise driven leaky IF neuron (Eq. (24)) that he/she refers to as our primary result (another severe misunderstanding on the part of the Reviewer as our main result is clearly Eq. (45-47)).

Argument III: The results are biologically irrelevant because there are neurons that fire much more irregular (in terms of the coefficient of variation) than considered in our paper

Thirdly, the Reviewer reiterates assertions that our approach and results are not relevant enough for publication in PLoS CB because the variability of the interspike interval in our model does not match the variability observed in neural data. Specifically, he/she writes:

> *3- Biological relevance*
>
> *Here I assume the theory is somehow correct due to unknown reasons for a LIF model in a specific parameter range and only discusses the manuscript's biological relevance. In response to my earlier concerns about the biological relevance, the authors provide an additional figure that shows the theory and simulations matches where the Coefficient of Variation (CV) is below 0.2-0.3. They also argued that this is a relevant range for the sensory systems.*
>
> *I need to admit that the suggestion of "Chacron et al. 2003" in my previous report was imprecise. I am sorry. I had in mind Fig.1 in Chacron et al. (JNeurosci, July 15, 2001) that the CV is about 0.2. Because in the original submission and revision, the input CV is about* $10^{-5} - 10^{-}3$ *(minimal noise). However, it is still unclear if the model's firing rate, when the ISIs CV is about 0.2, also matches with this data point by Chacron et al. (JNeurosci, July 15, 2001).*
>
> *There is enormous evidence that the CV in sensory systems can be much higher in contrast to what the authors claimed. To mention only a few examples: Locust auditory receptor neurons' data shows CV is above 0.5 (Schaette et al., JNeurophysio, 2005). Interestingly, Schaette et al. also report serial correlations in the data and indicate its functional relevance. In the early fly visual system, neurons can also have a very high CV of about 1.9 (see Fig.2 in Steveninck et al., Science 21, 1997). The high variability in spiking statistics of olfactory receptor neurons (ORN) is widely well known (see Duchamp?Viret et al. (JNeurobio 65, 2005), where it shows the CV range for ORN is 0.5-3.0).*
>
> *Therefore, the theory (if it is mathematically correct for some unknown reasons) is somewhat limited and cannot be applicable as general, as the authors claim in the abstract.*

Indeed, as the reviewer writes, variability of neural spiking is most often captured by the coefficient of variation (CV), i.e. the standard deviation of the ISI divided by the mean ISI, a relative non-dimensional measure of stochasticity of neural firing, that varies typically between 0.1-1.5 with occassional outlayers from this range to very high values (for bursting cells) and low values (for large neurons in vitro and for neurons in motor cortex). In his/her first report he/she initially cited Chacron et al. (who studied in the mentioned paper the P-unit of the weakly electric fish) to demonstrate that our theory would not account for realistic variability as measured by the coefficient of variation of the interspike interval. In the papers by Chacron on the P-units and in newer and more systematic explorations of the topic by Grewe et al. PNAS (2017) it is shown that the CV of the spontaneous activity in this specific sensory cell is between 0.1 and 1 (see Fig.1E in Grewe et al.). In the same more recent study, another type of sensory neuron is studied, ampullary receptor afferents, most of which have $CV < 0.15$. Indeed the P-units (the referee's example case from the Chacron paper) are considered very noisy and even their name (P-unit stands for probability unit), reflects the stochastic nature of their firing pattern. In our revised version we presented a new plot that demonstrated that our theory indeed accounts quantitatively for physiologically relevant range of CVs (up to 0.3) and qualitatively for even stronger variability (CVs up to 0.7). This covers the example by Chacron et al. (2001) and by Schaette et al. (2005) given by the reviewer, specifically, a large part of the relevant range for the P-units and quantitatively the entire range for ampullary receptors in the electrosensory system of weakly electric fish. Just to widen the view here: typical CVs of sensory cells are way below 1 (see e.g. examples in Brenner et al. PRE 2002) and most neurons in the motor cortex and many in visual and prefrontal cortex have CVs that are below 1 as well [see for a more recent study Mochizuki et al. J. Neurosci. 2016, Fig. 4 - their firing regularity parameter is related to the CV via $\log(\kappa) = -2\log(CV)$]. Of course, there are interesting exceptions or whole brain areas where the CV is higher because these cells are bursting and these cases are clearly *not* covered by our model and theoretical approach. Just to remind everybody what we said in our abstract regarding the applicability of our theory: *Assuming weak noise, we derive a simple formula for the serial correlation coefficient, a sum of two geometric sequences, which accounts for a large class of correlation patterns. The theory is confirmed by means of numerical simulations in a number of special cases including the leaky, quadratic, and generalized*

integrate-and-fire models with colored noise and spike-frequency adaptation. ... Finally, we also discuss the range of validity of our weak-noise theory. So we clearly state that we assume the driving noise to be weak and that we discuss the range of validity (hence, this range is not infinite because there is something to be discussed). We nowhere claim that our theory will cover correlation coefficients for *all kinds* of neurons, we are aware and we also make the reader aware of the limitations of our theory. Nevertheless, compared to everything else on the market, our approach seems to be by far the most general theory for serial correlations, still in the accessible form of a sum of two geometric series.

We also note, that the Reviewers approach appears as particularly problematic here, because we have managed to satisfy his/her demands to demonstrate that the theory is applicable for his/her specific example (sensory cells like the P-units with CVs about 0.2); in the second round the reviewer makes undue follow-up demands in order to discredit our work.

Appendix: Content of the papers cited by the reviewer and relation to our work

Our analytical approach in a nutshell

We consider a general integrate-and-fire model with adaptation driven by a combination white and colored noise. We perform a systematic linear-response approximation for the deviation of the interspike interval (ISI) from the mean ISI $\delta T_i = T_i - \langle T \rangle$. This allows us to calculate the variance and covariance of the ISI $\langle \delta T_i \rangle^2$ and $\langle \delta T_i \delta T_{i+k} \rangle$ which are consequently accurate up to second order. Eventually, we calculate the serial correlation coefficient (Eqs. (45-47) in the manuscript.) and the coefficient of variation (Eqs. (53-55)). In the following we discuss the cited papers and show that none of them have an additional contribution to the variance or covariance that is of second order.

Ermentrout et al. (J Comput Neurosci, 2011)

The reviewer writes:

> *Ermentrout et al. (J Comput Neurosci 31, 2011) calculate ISIs distribution's mean and variance for a general multi-dimensional oscillator dynamics with additive Gaussian noise. Their results presented on pages 20 and 21 under the Coefficient of variation are specific examples of this paper. This paper is not cited.*

Ermentrout et al. consider a phase model that is subject to a white noise $\xi(t)$ and delta kick at some time $\beta\delta(t-\tau)$

$$\frac{d\phi}{dt} = 1 + [\varepsilon\xi(t) + \beta\delta(t-\tau)]\,\Delta(\phi) + O(\varepsilon^2).$$

This choice is motivated by an experimental setup where the PRC of a real neuron is measured in a noisy environment. In this setup they calculate the phase dependent variance (page 188):

$$\mathrm{Var}(\tau) \approx \varepsilon^2 \left(\int_0^T \Delta^2(s)ds + 2\beta\Delta'(\tau)\int_0^\tau \Delta^2(s)ds - \int_\tau^{\tau+\beta\Delta(\tau)} \Delta^2(s)ds \right)$$

that contains the variance of the interspike interval in the simplest case of a noisy oscillator driven by white noise:

$$\mathrm{Var}_{\mathrm{ISI}} = \varepsilon^2 \int_0^T \Delta^2(s)ds.$$

This implies to leading order in the noise amplitude ε that the squared coefficient of variation reads

$$C_V^2 = \frac{\varepsilon^2}{T^2}\int_0^T \Delta^2(s)ds. \qquad (1)$$

Ermentrout et al. do not explicitly discuss the CV because the focus of their paper is on the variability of the PRC in a noisy setting.

We obtain the same result when we switch off both the colored noise ($\sigma = 0$) and adaptation ($\alpha = 0$) keeping only the white noise as an input. Hence, not our result is a special case of the result by Ermentrout et al. but quite the opposite, their above expression for the variance (or the corresponding formula for the CV, eq. (1)) is a special case of our general formula for a neuron with white and colored noise and adaptation (Eq. 53-55 in our manuscript.). The fact that our result agrees with that of Ermentrout et al. is another indication that our approach is not wrong as claimed by the reviewer under point 2. Given the opportunity to revise the paper once more, we would add the citation to Ermentrout for this reason.

Ota et al. (PLoS One, 2012)

The reviewer writes:

> *Ota et al. (PLoS One, 2012) calculated higher-order statistics of neural oscillators. Importantly, this work shows spike covariance (eq. 11), a related quantity to the authors' primary results in eq. 24, which must be evaluated up to higher orders in the perturbation expansion. These additional terms are missing in the authors' calculation (see point 2 in this report). This paper is also, unfortunately, not cited.*

In this paper Ota et al. consider a model perturbed by uncorrelated Gaussian noise (apparently a vector of independent noise sources)

$$\frac{d\mathbf{X}}{dt} = F(\mathbf{X}) + \sigma\xi(t),$$

perform the phase-reduction for sufficiently small noise intensities σ to obtain a first order approximation (in σ) of the phase dynamics

$$\frac{d\phi}{dt} = 1 + \sigma Z(\phi)\xi(t).$$

The authors then proceed to expand the deviation from the deterministic phase $\psi(t) = \phi(t) - t$ up to second order, which is methodological questionable (as it implicitly approximates the second order response function $Z_2(t_1, t_2)$ by $Z_1(t_1)Z(t_2)$, see, for instance, Eq. (7) in Ota et al.). They continue to define the n'th moment of the spike triggered ensemble (M$STE_n(\tau_1, ..., \tau_n)$) in Eq. (9), a statistics of the driving stimulus (conditioned on the activity of the driven system) for which it is unclear how it should be related to the interspike interval statistics. Furthermore, the model in the paper by Ota et al. would generate a renewal process and thus it would not be even meaningful to consider the problem of the calculation of ISI correlations for this model. Hence, it remains unclear why the reviewer thinks that this paper and its results are important for our paper.

Regarding the correct order of magnitude one has to take into account when computing the response of a nonlinear system to weak noise, it is interesting to follow the line of argument of the reviewer (and of Ota et al.) for a moment (though neither statistics nor model are of relevance for our work!). Ota et al. find that for the spike-triggered covariance (STC = M$STE_2(\tau_1, \tau_2)$ - M$STE_1(\tau_1)$ M$STE_1(\tau_2)$) the following approximation

$$STC(\tau_1, \tau_2) = \sigma^2\delta(\tau_1 - \tau_2) + \frac{1}{2}\sigma^4 Z'(t)Z(t)\delta(\tau_1 - \tau_2) + \sigma^4 H_{1/2}Z''(T - \tau_2)Z(T - \tau_1) + ...$$

It can be seen that they have to include higher-order terms if they do not wish to restrict themselves to cases where τ_1 and τ_2 are equal times (which would correspond to the variance of the spike-triggered average a certain time $\tau := \tau_1 = \tau_2$). This is so because they consider uncorrelated noise.

If we would be interested in computing their statistics for a model with colored noise, then the leading order would probably give another σ^2 term and this would be (especially compared to the σ^4 terms be dominant for sufficiently weak noise. It would be a valid approach to terminate the series after the second-order term and in the special case considered by Ota et al. we would prefer to do so because their higher-order correction seems to be doubtful because it does not involve the correct higher-order nonlinear response function but looks like a naive 'nonlinear' generalization of the linear response function.

Teramae et al. (Phys. Rev. Lett, 2009)

The reviewer writes:

In this paper, they have studied a general oscillator driven by colored noise. This is indeed similar to the model in eq. 2 of this submission. The result here indicates that a noisy oscillator phase's dynamics do have an order-one term orthogonal to the limit-cycle as well, see $k(\phi)$ in equation 12 therein, and therefore cannot be neglected.

Teramae et al. consider an interesting case where both the phase and amplitude are driven by a colored-noise.

$$\begin{aligned}\dot{\phi} &= 1 + \sigma h(\phi,\rho)\frac{y}{\sqrt{\tau_\eta}}\\ \dot{\rho} &= \frac{1}{\tau_\rho(\phi)}f(\phi,\rho) + \sigma g(\phi,\rho)\frac{y}{\sqrt{\tau_\eta}}\\ \dot{y} &= -\frac{y}{\tau_\eta} + \frac{\xi(t)}{\sqrt{\tau_\eta}}\end{aligned} \tag{2}$$

here $h(\phi,\rho)$ is the phase-amplitude response function. Now, the authors are not interested in the general colored-noise setting but they exclusively consider the limit of white noise $\tau_\eta \to 0$ which they connect with the limit of an infinitely fast amplitude relaxation ($\tau_\rho \to 0$). Their main interest is in studying how the ratio of the two vanishing time scales $\kappa = \tau_\eta/\tau_\rho$ affects the resulting phase dynamics. Again, no non-renewal behavior can be obtained in the white noise limit, no ISI correlations are present or discussed here, no colored noise and no adaptation plays a role in the results of this study. Nevertheless, we demonstrate that in eq. (3) the contribution of the amplitude-response onto the phase variable ϕ and in turn on the interspike interval is not of linear order in σ as claimed by the Reviewer.

The reviewer claims: *Teramae et al. (PRL102, 2009) clearly state that the orthogonal direction's fluctuation to the limit cycle remains order-one (see the text above the eq. 3 therein).* First of all, the Reviewer confuses different small parameters. Teramae et al. state that the amplitude ρ is of order $O(\varepsilon)$. This parameter ε however is related to the timescale of the colored noise $\varepsilon = \sqrt{\tau_\eta}$ because they are eventually interested in the limit where both the relaxation time of the amplitude τ_ρ, as well as the colored-noise time constant τ_η, simultaneously go to zero, so that $k = \tau_\eta/\tau_\rho$ remains constant. Secondly, Teramae et al. state that the *amplitude* $\rho = 0$ is asymptotically stable (below Eq. (2)) for which it follows that ρ is of linear order in σ in the stationary state (see below). The effect of the amplitude on the phase is mediated only by the phase-response function $h(\phi,\rho)$ which itself is multiplied by σ so that perturbations to the amplitude in order σ contribute in order σ^2 to the phase.

That said, we demonstrate explicitly that the consideration of the amplitude only affects the phase in second order, i.e. $O(\sigma^2)$, the actual small parameter that we are interested in. To this end we expand ρ and ϕ in terms of σ: $\phi = \phi_0 + \sigma\phi_1 + \sigma^2\phi_2 + O(\sigma^3)$ and $\rho = \rho_0 + \sigma\rho_1 + O(\sigma^2)$. Sorting ϕ in orders of σ yields

$$\begin{aligned}\dot{\phi}_0 &= 1\\ \dot{\phi}_1 &= h(\phi_0,\rho_0)\frac{y}{\tau_\eta}\\ \dot{\phi}_2 &= \left(\phi_1\left.\frac{\partial h(\phi,\rho)}{\partial\phi}\right|_{\phi_0,\rho_0} + \rho_1\left.\frac{\partial h(\phi,\rho)}{\partial\rho}\right|_{\phi_0,\rho_0}\right)\frac{y}{\sqrt{\tau_\eta}}.\end{aligned}$$

For ϕ to be affected by the noise filter through the amplitude dynamics in order $O(\sigma)$ that would require $\rho_0 \neq 0$ (see the dynamics for ϕ_1). The different orders of the amplitude ρ in turn are determined by:

$$\begin{aligned}\dot{\rho}_0 &= \frac{f(\phi_0,\rho_0)}{\tau_\rho(\phi_0)},\\ \dot{\rho}_1 &= g(\phi_0,\rho_0)\frac{y}{\tau_\eta} + \phi_1\frac{\partial}{\partial\phi}\left.\left(\frac{f(\phi,\rho)}{\tau_\rho(\phi)}\right)\right|_{\phi_0,\rho_0} + \frac{\rho_1}{\tau_\rho(\phi_0)}\left.\frac{\partial f(\phi,\rho)}{\partial\rho}\right|_{\phi_0,\rho_0}.\end{aligned}$$

Because the limit cycle is stable and we are interested in the stationary case, the term $\rho_0 = 0$ vanishes because it corresponds to the case where the system is unperturbed, i.e. $\sigma = 0$ and, as Teramae et al. clearly state, "*without loss of generality, we can shift the origin of ρ to $\rho = 0$ on the limit cycle solution* and *[...] the limit cycle at $\rho = 0$ is stable, [...]*. Put differently, since the limit cycle is stable, the leading order of ρ is indeed σ. The only exception to this is if one would be interested in transient effects induced by initial condition

off the limit cycle, which we are not because we consider stationary spike trains. Substitute the expansion of $\rho(t)$ into the differential equation that governs the phase dynamics yields

$$\dot{\phi} \approx 1 + \sigma h(t + \sigma\phi_1(t), \sigma\rho_1(t)) \frac{y}{\sqrt{\tau_\eta}}$$

h can be approximated for small σ as follows: $h(\phi, \rho) \approx h(t, 0) + \sigma\phi_1(t)\frac{dh(t,0)}{d\phi}\frac{y}{\sqrt{\tau_\eta}} + \sigma\rho_1(t)\frac{dh(t,0)}{d\rho} + O(\sigma^2)$ which results in

$$\dot{\phi} \approx 1 + \sigma h(t, 0)\frac{y}{\sqrt{\tau_\eta}} + \sigma^2\phi_1(t)\frac{dh(t, 0)}{d\phi}\frac{y}{\sqrt{\tau_\eta}} + \sigma^2\rho_1(t)\frac{dh(t, 0)}{d\rho}\frac{y}{\sqrt{\tau_\eta}} + O(\sigma^3) = 1 + \sigma h(\phi, 0)\frac{y}{\sqrt{\tau_\eta}} + O(\sigma^2) \quad (3)$$

here $h(\phi, 0) = Z(\phi)$ is the phase-response curve. Note that the terms ϕ_1 and ρ_1 are chosen so that they are $O(1)$. Thus, without considering the particular result obtained by Teramae in Eq. (11) and (12) it can be seen that the phase dynamics are, in leading order of σ, determined by the phase-response curve. Of course, Teramae et al. obtain qualitatively the same result in their particularly chosen white-noise limit: In their Eq. (12) $\dot{\phi} = 1 + \sigma^2\frac{Y(\phi)}{1+k(\phi)} + \sigma Z(\phi)\xi(t)$ where the contribution of the amplitude to the phase is proportional to the term $Y(\phi) = h_0(\phi)g_1(\phi)/2$ which is clearly of higher than linear order and will contribute to changes of the single ISI to second order only. It is absolutely unclear to us why the Reviewer conclude that "*the result here indicates that a noisy oscillator phase's dynamics do have an order-one term orthogonal to the limit-cycle as well*". In fact, the result by Teramae et al. indicate: Perturbations to the amplitude of order $O(\sigma)$ contribute in order $O(\sigma^2)$ to the phase.

van Vreeswijk (PRL84, 2000)

The reviewer writes:

> *I end this list with a paper by van Vreeswijk (PRL 84, 2000). In that paper, van Vreeswijk shows in a general setting, as a result of a perturbation, the timing of n-th spikes has a higher-order contribution from the corresponding Floquet exponent (eq. 6-9). These contributions are essential for calculating the interspike-intervals serial correlations. Moreover, this work includes the specific case of spike-triggered-adaptation, and it has also derived the additional contributions in perturbation series (see eq. 18 and 19 therein). The work here is highly relevant for authors, but it is overlooked in Ramlow and Lindner's revision.*

The paper by Van Vreeswijk addresses the asynchronous neural spiking in a recurrent network of *strongly coupled* neurons. We have a good understanding of such network problems as proven by recent papers from our group (see, for instance, Dummer et al. Front. Comp. Neuroscie. 2014 and Van Meegen & Lindner Phys. Rev. Lett. 2018, Vellmer & Lindner Phys. Rev. Research 2019), however, this kind of problem is fundamentally different from what we consider in our paper. Van Vreeswijk studies the mean-field theory for a network of noiseless units with strong input, not a single neuron with weak noisy input as is the subject of our study. Van Vreeswijk is interested in the stability of the asynchronous state of this network and not at all in interspike interval correlations (in fact, all his units will generate a perfectly regular spike train even if all of them are perfectly desynchronized - hence, ISI correlations as well as ISI variance is not an appropriate description here). Through the strong coupling of the oscillatory units in the network the pure original phase description of the single oscillator will nor be applicable anymore or only in a modified form that takes (through the mean-field theory) also the amplitude dynamics into account. However, we are only concerned with a weak external driving of the oscillator, for which Van Vreeswijk in his introduction concisely summarizes:

> *A special case in which much theoretical progress has been made is in the case of oscillators with weak interaction. These systems can be studied using perturbation theory: One first characterizes the units by analyzing how they react to small transient perturbing input in the absence of interactions, and then closes the system by relating these perturbations to the activity of the other cells [5,6]. Because the interactions are weak,* ***perturbations in directions orthogonal to the limit-cycle trajectory can be neglected.*** *This results in a system in which the units are described by a phase variable that denotes their position on the limit cycle, and the interactions depend only on their phase differences.*
> (Highlighting of certain phrases are by us!)

This weak-input case is exactly what we are concerned with in our paper, and the subsequent mean-field analysis of *strongly coupled* units in a network (including the Floquet framework and the special case of the adapting IF neuron) is of no use for our problem.

If the reviewer would have read the paper by Van Vreeswijk carefully, he/she would have noticed the passage we quoted above which is in line with what we said in our first reply: for weak input noise (which replaces weak network input in our case), the phase description suffices (e.g. for the computational problem of calculating analytically the ISI correlations). Hence, citing the PRL by Van Vreeswijk looks like another attempt to discredit our analytical approach by a flawed comparison to a theory devoted to an entirely different problem.
