# Supplementary material for: Interspike interval correlations in neuron models with adaptation and correlated noise
Source: PLoS Comput Biol. 2021 Aug 27;17(8):e1009261. doi: 10.1371/journal.pcbi.1009261 (PMC8428727; doi:10.1371/journal.pcbi.1009261)
Supplement: Attachment — Submitted filename: reply_letter.pdf [file pcbi.1009261.s003.pdf]

PLOS Computational Biology Submission
PCOMPBIOL-D-20-00911
Interspike interval correlations in neuron models with adaptation and correlated noise

Reply to the editor's and the reviewers' comments

Dear Prof. Gershman, dear Prof. Ermentrout,

thank you very much for your time and efforts devoted to our manuscript. We have substantially revised our ms to take into account the critical remarks of the third reviewer and hope that the paper can now be accepted for publication in PLoS Computational Biology.

Sincerely yours,
Lukas Ramlow and Benjamin Lindner

Editor

I have received reviews that address the current version of your manuscript and like the first round, they are mixed. Two ask for minor revisions. The third makes strong objections based on some of the material in the introduction in which the authors seem to imply that cortical variability is the same mechanism as single neuron variability, a point that is certainly not true. Thus, I think in the revised version of the paper, the authors should play this aspect down and furthermore convince the first reviewer that this work is relevant to networks of neurons. This may be a tall order and I hope the authors will consider it.

Thank you for this concise summary. Details of how we addressed the criticism of the first reviewer will follow below; here only briefly : (i) in the revised manuscript we have stressed once more that interesting spiking statistics is obtained also outside of cortex and beyond recurrent networks and we emphasize that the main field of application of our theory is the sensory periphery; (ii) we also point out that even in recurrent networks there are scenarios, in which our theory is capable to describe rich spike statistics (mean-driven neurons driven by recurrent network noise) - we have extended the theory to include one such case in the paper. We hope that this convinces the reviewer and the editors that (a) we do not intend to oversell our results and (b) they can be useful to understand spike statistics in various non-trivial situations.

Please also note that the suggestion of the third reviewer to test our theory for a conductance-based model has led to yet another extension of our framework - we now apply our theory to a stochastic version of the Traub-Miles model with a slow potassium current which mediates spike-frequency adaptation. We find excellent agreement of the theory with numerical simulations of this conductance-based spiking neuron model.

In response to the reviewer comments we have thus substantially extended our results and hope that this will be appreciated by all reviewers.

Reply to Third Reviewer

In "Interspike interval correlations in neuron models with adaptation and correlated noise" by Ramlow and Lindner, the authors develop a theory of how spiking models with thresholds and voltage (+aux vars) reset behave when a neuron is tonically firing subject to weak noise. In this and several of their prior papers, they analyze a quantity called the serial correlation coeff (SCC), which is the trial-average of interspike intervals (ISI) that are separated k apart (k=0 <=> variance of ISI). The novel contribution is they have extended prior work to now include 3 contributing effects: white noise, adaptation current and correlated (in-time) noise, the later 2 being the key because they are combined. Their prior works and other works only considered colored noise or adaptation separately. The work is certainly technically sound: taking integrals, manipulating various terms to get SCC, series expanding and working in the Fourier domain (as far as I can tell).

But I do not believe this paper warrants publication in PLOS CB. I write this with angst because I hold the works of these authors (Lindner) in the highest regard, here are reasons for my personal opinion.

We thank the Reviewer for his/her time, effort, and critical input that has forced us to develop another application for our theory and we hope that we have clarified all issues raised by this reviewer in a convincing manner.

Major comments

1. *Abstract: ".. computational studies have identified spike-frequency adaptation and correlated noise as the two main mechanisms.." The authors argument that rich statistical spiking structures exist and need to be explained is valid, but I find it very hard to believe that a single cell setting (or statistically identical population) can realistically model spiking data — I'm not sure what specifics they have in mind but the paper reads as if these methods apply in general. Aren't the network structures (coupling, balanced inhibition, prob of connections) and changes in state (top-down modulation) among the key driving forces that result in the rich spiking dynamics? Is there experimental evidence to show that an uncoupled single neuron endowed with adaptation currents subject to white and correlated noise is the same as brain recordings?*

   We see that by too vague formulations, we may have triggered the wrong associations in this reviewer's mind that our theory is applicable to all kinds of complex spiking patterns, particularly in cortex.

   Let us start our reply with a (somewhat trivial) disclaimer: a large part of the rich statistical spiking structure cannot be addressed with our analytical method, namely, spiking that must be regarded as a non-stationary process (which, admittedly, excludes many interesting cases). The ISI correlation coefficient is a stationary statistics that applies only to stationary processes, and that is what we focus on. When do we deal with quasi-stationary processes? This is for instance the case for spontaneous firing in both the sensory periphery but also in higher-brain regions, e.g. in the so-called asynchronous irregular state (see e.g. Renart et al. Science, 2010).

   When you look at the literature for ISI correlation coefficients, most examples are from the sensory periphery, e.g. auditory or electrosensory neurons, cells that are *not* subject to recurrent input or lack even any synaptic input. Although these cells have a simple dynamics (often they fire in a mean-driven fashion), the presence of correlated noise input, correlated channel noise, and adaptation can cause rich stationary spike statistics, which can be captured by our theory. Special examples of peripheral neurons have been studied in our group in collaboration with experimenters (see e.g. Fisch et al. J Neurosci 2012, Bauermeister et al. PLoS CB 2013, Schwalger et al. J Comp Neurosci 2015), which provides from our perspective plenty of motivation for the general unifying theory provided in our paper.

   Now, the reviewer seems to be more interested in the spiking statistics of cortical neurons and asks how the input from a network can be captured by a single-neuron-plus-colored-noise setting. He/she wonders whether the complex spike statistics is rather due to the elaborate connectivity of neurons but not due to some input noise. However, it is the central idea of stochastic mean-field theory that complex network input (in stationary situations such as the AI state) can be approximated as a stochastic process, i.e. a *correlated (colored) network noise.* The type and strength of the connections, the synaptic (short term) dynamics, the effect of neuromodulators, the heterogeneity of cells in the network will all affect the shape of these input correlations and they, in turn, will affect the spike statistics of the driven cell. In a recurrent network, moreover, the driving input fluctuations (i.e. the network noise) will also depend on

the generated spike-train correlations in a self-consistent manner (this was early addressed by Lerchner et al. 2006 but explored more recently in our research group, see e.g. Dummer et al. Front Comp Neurosci 2013, Van Meegen & Lindner Phys. Rev. Lett. 2018, Vellmer and Lindner Phys. Rev. Res. 2019). This means that in general the correlations of the network noise are not known. However, from some experiments (Bair et al J Neurosci 1993) and from numerical and analytical studies (e.g. Vellmer and Lindner Phys. Rev. Res. 2019) it is known, that this network noise has often a green spectrum (a spectrum that has a dip at low frequencies and is otherwise flat; the name by the way is borrowed from the notion of complementary colors, i.e. green = white minus red). We furthermore know that some neurons even in cortex (which is otherwise dominated by excitable cells) are mean-driven (the fraction of neurons with low CV). We can modify our theory to include also this green noise and study how a green noise affects a tonically firing neuron (potentially also endowed with adaptation). We have included this case in the new section **Adaptive leaky integrate-and-fire model with network-noise-like fluctuations** as one application of our theory to a cortical cell. We have, however, also pointed out again the limitations of our theory (i.e. that it cannot be applied to excitable cells!). We hope that this Section together with the now demonstrated applicability of our theory to conductance-based models does convince the Reviewer that our method is indeed relevant for a broad range of neurons, even to special cases in cortex.

We have pointed out the main field of application for our theory and the complications in the recurrent-network case in the introduction on p. 3 as follows (additions in text are in red):

> In this article we extend the *weak* noise theory developed by Schwalger and Lindner [9] for mean-driven neurons to include multiple correlation-inducing processes. Our theory mainly applies to noisy neurons in the sensory periphery, in which the type of noise and the adaptation mechanisms are known. Cortical neurons, on the other hand, are more difficult: they typically operate in an excitable firing regime and the network noise that drives them is generally not known with respect to its statistics; below we discuss a special case of cortical firing that can nevertheless be captured by our theory.

We also point out the one cortical example that we have studied in the abstract:

> ... we demonstrate that our theory can account for a nonmonotonic dependence of the correlation coefficient on the channel's time scale. Another application of the theory is a neuron driven by network-noise-like fluctuations (green noise). We also discuss the range of validity of our weak-noise theory ...

2. *Author Summary: "As these correlations can significantly improve information transmission and weak-signal detection, it is an important task to develop analytical approaches to these statistics for well-established computational models". This is not convincing. Why do we need analytics for this class of models specifically for coding (info trans & signal detection)?*

   What specifically is it about the simulations that are not helpful for these coding questions? Is there an application of these formulas that could be tested experimentally (and/or can we use these formulas to suggest future experiments) that one would not have without simulations? I'm not specifically asking for actual data to validate the theory, but I feel like this is oversold.

   The simplest answer to the question of how important ISI correlations are for coding is certainly given in the papers by Ratnam and Nelson J. Neurosci. (2000) for the spike count variability: the long-term Fano factor (variance of the count over its mean for a long time window) depends crucially on the coefficient of variation (C_V) of the single ISI but also on the correlation coefficients

$$\lim_{T\to\infty} F(T) = C_V^2(1 + 2\sum_{k=1}^{\infty}\rho_k).$$

   (a very similar formula for the power spectrum of the spike train is already discussed in the paper).

   The Fano factor in turn determines the noise in a signal detection task; a reduced Fano factor (as can be achieved by a negative sum of the correlation coefficients in the above formula) implies reduced noise and thus improved detection. Remarkably, correlation coefficients in electrosensory receptor cells, so

called P-units, in the weakly electric fish have different shapes of correlation coefficients but their sum is always close -1/2 (the value that would make the Fano factor vanish completely).

More generally, it has been shown in analytical comparison of renewal and nonrenewal spiking models that the mutual information about a broadband stimulus is systematically (i) increased for negative ISI correlations (Chacron et al. *Phys. Rev. Lett.* 2004, Lindner et al. *Phys. Rev. E* 2005) and (ii) decreased for positive ISI correlations (Blankenburg and Lindner *Math. Biosci. Eng.* 2016). Mechanisms for these beneficial and detrimental effects of serial ISI correlations have been discussed in review papers (Avila-Akerberg and Chacron *Exp. Brain Res.* 2011, Lindner EEE Trans. Mol. Biol. Multi-Scale Commun. 2016).

Now given the importance of serial ISI correlations for coding, it is beneficial to relate them by formulas to cellular properties of the model (encapsulated in the PRC), to properties of the adaptation and to the properties of the noise. This is what our theory provides. Of course, you can inspect these models and their coding properties by simulations; we have nothing against simulations but use them frequently too. However, for questions like: 'What kinds of correlations are possible?' or 'What determines the sign of the ISI correlations?' our theory is useful and predictive, as we demonstrate in the paper for many different examples.

In order to highlight the importance of the SCC for coding and signal detection, we have somewhat extended the corresponding part in the discussion and now write on p. 18

> Having a reduced background spectrum at low frequencies (due to negative ISI correlations) may enhance the signal-to-noise ratio in this frequency band (but also diminish it in other bands) [3, 4]; positive correlations, on the contrary, may diminish the mutual information about a broadband stimulus [14]. A similar argument for the beneficial effect of negative ISI correlations can be made for a signal detection task [1] by means of the asymptotic Fano factor $F(T) = \langle (N(T) - \langle N(T) \rangle)^2 / \langle N(T) \rangle$ (where $N(T)$ is the spike count in the time window $[0, T]$) , that is simply related to the low-frequency limit of the power spectrum stated above by $\lim_{T\to\infty} F(T) = S(0)/r_0$. For the transmission of time-dependent signals, interval correlations are a way to control the noise spectrum and thus to shape the information transmission in a frequency-dependent manner, contributing to what is known as information filtering [15]. In all these cases, our theory describes how exactly the cellular dynamics (PRC), the adaptation (strength and time scale) and the noise (strength and correlation time) affect the serial correlations and by them the signal transmission properties of the respective neuron.

3. *I am impressed that even with all of their assumptions they can get particular excellent matches to the simulations. Does the assumption that the period is on average T^* (the same as the 0 noise case) really hold throughout all of these parameters? Is checking this consequential for the theory? I thought it would be easily violated with strongly correlated noise and/or strong adaptation.*

   The assumption that the mean ISI is well approximated by the deterministic ISI does hold throughout all parameters that were considered. We have not excluded any particular parameter values. In general the assumption $T^* \approx \langle T \rangle$ is well met if the considered model is mean driven (the further from the bifurcation point the better). Furthermore, the suspicion that strongly correlated noise would easily violate the aforementioned assumption might be wrong. It is certainly true that long correlated noise will increase the chance to observe a interspike intervals that deviate considerably from the mean (meaning more strongly than in the case of short correlated noise). This however holds true for both positive and negative deviations from the mean and hence affects the variance but not necessarily the mean. This can also be seen in Fig. 1 where the variance (left panel) increases with τ_η while the mean (right panel) is mostly unaffected. Generally we confirm what is known from the literature, i.e. that mean ISI (or period) of an neuron model (oscillator) is not changed considerably by correlated noise. See for instance Galan [6] who used a PRC-based approach to calculated the mean change of an oscillators frequency with respect to the correlation time of the driving Ornstein-Uhlenbeck process.

4. *Although these calculations are interesting to a subset of the compneuro community, I believe they are better suited for another journal (e.g., Physical Review Letters or Physical Review X, or many SIAM journals) where the specific absence of cortical function or application to a particular neural system would be better received by readers.*

In the revised version of the manuscript we have devoted some effort to apply our theory to even more examples than before: the case of network-generated noise acting on a tonically firing neuron in cortex (with or without adaptation) and the case of a conductance-based neuron model with adaptation and noise. Together with the already discussed case of adaptation-channel noise, this should demonstrate that our theory is indeed relevant to a a variety of important biological situations.

5. *I can see the qualitative connections between serial ISI (scaled) for describing spike stat dynamics, but how is it related to spike counts that tell us something about coding? There is a blurb on lines 148-149 that says there is a connection between this and Fano Factor, and they cite Ref [21]— I assume Fig 5 in Ref [21] is the connection. I think [21] describe how larger or smaller values of serial ISI are related to smaller Fano Factor, which in that study is where maximal gain of discriminability occurs. This is important scientifically, but I believe the value of precise serial ISI calculations is pretty far removed from spike stats that effect coding/decoding.*

   As we have pointed out above already, the Fano factor, given by $F(T) = \langle \Delta N^2 \rangle / \langle N \rangle$ (variance over mean of the spike count $N(T)$ in the time window $(0, T)$) is related to the serial correlation coefficients by $\lim_{T\to\infty} F(T) = C_V^2(1 + 2\sum_{k=1}^{\infty} \rho_k)$. In signal detection theory, the Fano factor plays an important role e.g. in the lower bound to the Fisher information (see e.g. Stemmler *Network: Comp. Neural Syst.* 1996), which can be written as follows:

$$J_{LB} = \frac{\partial \langle N \rangle / \partial \mu}{F \cdot \langle N \rangle}$$

   where μ is the input current. So, the Fisher information increases when the Fano factor is decreased (it also depends on the mean spike count and its dependence on the input current though).

   Another example is given by a neuron driven by a weak and low-frequent periodic signal $\varepsilon \cos(\omega_s t)$, the transmission of which is characterized by the signal-to-noise ratio (SNR) which can be written as

$$SNR \propto \varepsilon^2 \frac{|dr_0/d\mu|^2}{r_0 F}.$$

   Again, a low value of the Fano factor will be beneficial - it leads to a high SNR.

   From $\lim_{T\to\infty} F(T) = C_V^2(1 + 2\sum_{k=1}^{\infty} \rho_k)$, it is evident that the asymptotic Fano factor is not only affected by the first serial correlation coefficient but *by all of them.* That is why formulas that relate the SCCs at all lags to the properties of neuron (PRC), adaptation, and noise can help us to understand the coding properties of neurons.

6. *How does the theory vs sims plots look for just the numerator of eq (1), $\langle T_i T_{i+k} \rangle$? That would have been interesting to see if the net effect of the accuracy is because both num and denom are accurate or is something washed out in the normalization. I think the pieces are there (eq (34)).*

   We demonstrate here that the accuracy of the theory is no coincidence in a sense that the numerator and denominator deviate similarly from the "true" value that is obtained from simulations. To this end, we consider a LIF model driven by colored and white noise. The covariance $\langle T_i T_{i+1} \rangle$ and variance $\langle T_i^2 \rangle$ are readily obtained from Eq. (34) in this case (we have chosen the covariance at lag $k = 1$ because deviations should be most pronounced here).

   Even though, it is certainly educational to display the covariance to convince the Reader of the theory's accuracy, we would like to avoid to show another statistics in our manuscript. Simply because ρ_k is a convenient, widely accepted measure for the correlations between ISIs while the covariance $\langle T_i T_{i+k} \rangle$ is not.

7. *I definitely liked the expositions around figs 4,5,6,7 (e.g., lines 185-206), they are technical but they handled the dynamics well. Fig 8 is also key to see when the weak noise starts to break down, although the deviations from simulations are small.*

   Thank you for appreciating our efforts to handle the presentation of our results in a comprehensible way.

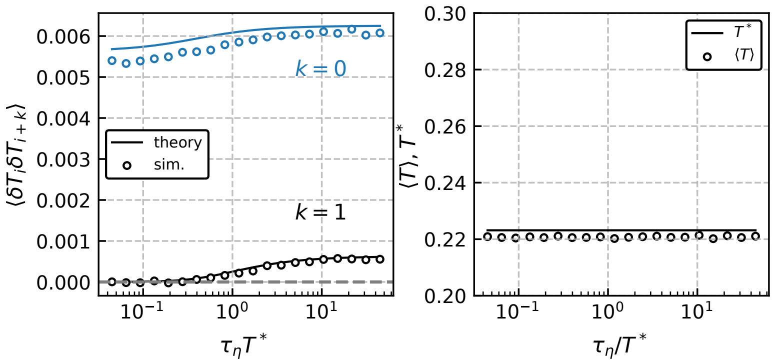

Fig. 1 – Comparison between $\langle T_i T_{i+1} \rangle$ and $\langle T_i^2 \rangle$ (left) as well as $\langle T \rangle$ and T^* (right). The output variability is rather high for our weak noise theory, i.e. $C_V \approx 0.3$. Parameters: $\gamma = 1$, $\mu = 5$, $D = 0.25$, $\sigma^2 = 0.25$.

Minor comments

1. *Eq (2b) and line 77; is it that common to have the aux vars w reset upon spiking? I feel like that is very artificial, not commonly done in any biophysical models I have seen.*

   To the best of our knowledge resetting the auxiliary variable in a GIF model has been used before in several publications (see Jolivet et al. *J. Neurophysiol.* 2004; Engel et al. *J. Neurophysiol.* 2008, Blankenburg et al. *J. Comput. Neurosci.* 2015). In Blankenburg et al. 2015 there is also a comparison made to a two-dimensional spiking model (the Morris-Lecar model) that motivates this reset rule for the auxiliary variable. What this means is that w does not carry memory about previous spike times so that the spike-train (in the absence of a slow adaptation current and colored noise) is a renewal process. This is indeed the case for most conductance-based models according to our experience. Take for instance the Traub-Miles model that we now consider at the end of the Results section. If the model is not subject to a slow adaptation current or correlated noise but only to uncorrelated noise, it does not exhibit significant ISI correlations. A simplified spike generator, as the GIF model, considered in the same setup should thus also not exhibit ISI correlations. This is ensured by resetting the auxiliary variable.

   We have added a sentence on p. 4

   > Note that the fixed reset of the voltage and auxiliary variable ensures that the spike-train is renewal in the absence of these two slow processes.

2. *line 342 "permits in most cases only numerical solutions" Don't the formulas in eqs (3)—(6) require numerical calculations of integrals and various functions? How about the PRC Z (even in eqs (7), (8), (10)), and the adjoint method is mentioned in Methods section, which has to be solved numerically. I guess the authors mean they can 'transparently' see how parameters effect SCC.*

   What we meant is that the Fokker-Planck framework rarely allows for analytical insides in a sense that, as the Reviewer said, we can transparently see how for instance the adaptation and colored noise affect the general correlation coefficient. We have thus reformulated on p. 16

   > The most general theory of these statistics uses the framework of a multidimensional Fokker-Planck equation [44, 76, 77] that, however, permits in most cases only numerical solutions that do not permit simple conclusions on how certain parameters shape the SCC.

3. *lines 411-412 ", we may consider ISI correlations for neurons connected in the large recurrent networks of the brain", but the theory here assumes the firing rate is constant (line 473) and the noise is weak; what large recurrent networks in the brain have these assumptions?*

   Our discussion here tries to give motivation why the serial correlation coefficient is interesting, irrespective of the question whether we can analytically calculate it. However, we see that this might be confusing and interpreted in the way of the reviewer, namely that we claim that our theory can be

without any restrictions applied to cortical neurons. We have now reformulated this paragraph to make clear that our theory cannot in general be applied to cortical cells - with the notable exception of the example that we study in the paper.

On p. 18 we now write in the discussion:

> Thirdly, we may consider ISI correlations for neurons connected in the large recurrent networks of the brain. In networks correlations can be evoked by the slow or oscillatory (narrow-band) noise emerging from the nonlinear interactions of neurons [8, 11, 13, 16, 17], by deviations of presynaptic spike statistics from Poisson statistics [10, 12], but also by synaptic short-term plasticity [12]. As outlined above for the case of a single noisy neuron, such ISI correlations can likewise affect the signal transmission properties of whole populations [17]. Moreover, the specific strength and pattern of ISI correlations may be informative about properties of the network (again in combination with other statistics). Although our theory cannot be readily applied to cortical neurons (many cells are excitable and the driving fluctuations are strong), it can be used in certain special situations, as we have demonstrated here.

Reply to Fourth Reviewer

Understanding the non-renewal nature of neural spike trains is a problem of long-standing and historical interest in theoretical and systems neuroscience. In recent years researchers, Prof. Lindner's group prominent among them, have devoted a great deal of effort to develop tools to link the mechanisms that make neural activity non-renewal to measurable statistics of that activity.

Ramlow and Lindner present calculate the serial interspike interval correlations for multi-dimensional integrate-and-fire neurons in a tonically firing regime with 1) spike-triggered adaptation, 2) weak white and 3) weak colored noise. While the impact of each of those three features on ISI correlations had been studied separately, their interplay has so far eluded description. By showing how the combination of these mechanisms can give rise to qualitatively different ISI serial correlation functions than either mechanism alone, this paper takes an appreciable step towards linking these theoretical studies with experiments.

The authors have convincingly rebutted the comments of reviewer 2 from the previous round and I agree with their rebuttal. I believe this paper is thus a solid theoretical contribution, meriting publication in Plos Comp. Biol.

We thank the Reviewer for the appreciation of our work and the accurate description of the content of our manuscript.

Major comments

1. *The authors described equation 3, their central theoretical result, as a linear combination of the isolated ISI correlations $\rho_{k,a}$ and $\rho_{k,\eta}$. But $\rho_{1,\eta}$ appears in the coefficient A, and the product $\rho_{1,\eta}\ \rho_{1,a}$ appears in the coefficient C. So isn't this a nonlinear combination of the isolated serial correlation coefficient functions? Describing this as just as a linear combination of $\rho_{k,a}$ and $\rho_{k,\eta}$ seems to unfairly neglect its complexity.*

   We agree with the Reviewer that the particular statement in line 129, namely *"Our main result Eq. (3) implies that the SCC for a general stochastic IF model with both adaptation and correlated noise is in fact a linear combination of the specific SCCs Eq. (5) and (6), i.e. it is a sum of two geometric sequences."* is somewhat misleading and can be formulated more precisely. What we had in mind is what was described in line 123, namely that *"the dependence on the lag k is carried exclusively by the two specific SCCs $\rho_{k,a}$ and $\rho_{k,\eta}$."* On p. 6 we have reformulated our statement as follows:

   > Our main result Eq. (3) implies that the SCC for a general stochastic IF model with both adaptation and correlated noise is in fact a linear combination of two geometric series. These geometric series are determined by the two correlation-inducing processes and agree with the specific SCCs Eqs. (5) and (6) except for a constant prefactor (constant with respect to the lag k).

Minor comments

1. *Throughout the paper the authors reference Type 1/ Type 2 neurons (and also Type 1/ Type 2 PRCs, which they describe). I for one had forgotten the definition of Type 1 / Type 2 neurons, and would appreciate a reminder the first time they are used in the paper.*

   We have added such an explanation on p. 3:

   > We relate statistics of the spike train, namely the SCC ρ_k to intrinsic properties of nonlinear neural dynamics captured by the phase-response curve (PRC). The PRC measures the shift of the next spike time of a neuron subject to a small perturbation at different times in the firing cycle [39–41]; see Fig. 1 for an illustration of the method. The shape of the PRC depends crucially on the neuron type: type 1 neurons which bifurcate from a quiescent to a tonically firing regime via a saddle-node on invariant circle bifurcation possess a purely positive PRC, whereas type 2 neurons which undergo a a supercritical Hopf bifurcation have a partially negative PRC (see [5, 7] for more details on these neuron types). Our theory applies to both neuron types and predicts qualitatively novel patterns of interval correlations that deviate from a single geometric sequence; such deviations have been recently reported experimentally ...

2. *Lines 89-92: This sentence is devoted to describing a variable defined for convenience in intermediate calculations in the methods section. u(t) doesn't appear in the subsequent equations of the results section so talking about it here confused me. To unify the text with figure 1, I would suggest defining u here.*

   We reformulated the passage to emphasize that $u(t)$ is a general perturbation (from the limit cycle) that eventually determines the correlation coefficient because it carries memory about previous activity; we give now also a reference to the explicit definition of $u(t)$ later in the ms. On p. 4 we now write:

   > The presence of both white and colored noise will affect the voltage dynamics directly. However, there is also an indirect effect through noise-induced deviations of the adaptation variable from the deterministic limit cycle. As outlined in the method section, the combined effect of the direct and indirect perturbations on the next spike time is subsumed in the perturbation function $u(t)$ that measures the deviation from the deterministic limit cycle and is shown exemplary in Fig. 1 (for the detailed definition of $u(t)$ see eq.(35)). This function typically attains both positive and negative values and carries memory about previous activity and stimuli.

3. *Equation 4d: The variable a^*, on the right-hand side of $\nu = \ldots$, is not defined here and the definition is hidden much later in the methods section, hard to find from this early point of the results.*

   Thank you for the careful examination of our manuscript. We have added a line on p. 6

   > ... and deterministic peak adaptation value $a^* = (\Delta/\tau_a)/(1 - \exp[-T^*/\tau_a])$. Remarkably, the dependence on the lag k ...

4. *Line 466: Does the adjoint method provide a route to calculate the phase response to perturbations in the auxiliary or adaptation variables?*

   Yes, it does. In the Section **Adjoint Method** we have explained the general method. In Eq. (12) $\mathbf{Z}(t)$ is a vector that contains the phase response functions in all directions. For the GIF model, for instance, the PRCs in all three directions can be found by solving the following system of linear differential equations (the index denotes the direction or variable to which the perturbation is applied):

   $$\frac{d}{dt}\begin{pmatrix} Z_v(t) \\ Z_w(t) \\ Z_a(t) \end{pmatrix} = \begin{pmatrix} \gamma & -\tau_w^{-1} & 0 \\ \beta & \tau_w^{-1} & 0 \\ -1 & 0 & \tau_a^{-1} \end{pmatrix} \cdot \begin{pmatrix} Z_v(t) \\ Z_w(t) \\ Z_a(t) \end{pmatrix} \tag{1}$$

   We highlight this fact now explicitly on p.19

   > Each component quantifies the linear response of the spike time to a perturbation of the limit cycle in the corresponding variable of the model. In particular, this implies that we can also calculate the phase-response with respect to perturbations of the auxiliary or adaptation variables.

Reply to Fifth Reviewer

Major comments

1. *I follow the argument of the authors, that they cannot cite all related papers. To my understanding reviewer 2 had pointed out these specific papers to underline on his major critics (see 4 below)*

   Our understanding was that reviewer 2 complained about the absence of certain references but this might be a matter of interpretation.

2. ***Biological relevance, significance, novelty, readership.***

   Spike-triggered cellular adaptation is ubiquitous in spiking neurons across systems in vertebrate and invertebrate species. Equally, colored noise is evident in neurons. Both influence output spike/interval statistics. The serial correlation of inter-spike intervals is a wide-spread phenomenon and has implications for information transmission and response reliability, this functional relevance is nicely covered in the Discussion section of the manuscript.

   The treatment of both, colored noise and SFA, and the derivation of serially correlated intervals is, to my knowledge, new. I think this is also understood by reviewer 2 who does not dispute the novelty of the manuscript. Describing the causal relation mathematically and with a claim of generality makes a significant contribution.

   PLoS CB aims at a broad readership. The present MS is certainly heavy to digest, too heavy for most of a broad readership. However, I believe that theoretical manuscripts like this need to have their place in PLoS CB if the mathematically treated problem is of broader relevance, which is given here because adaptation and input noise are fundamental aspects of how single neurons compute in a network.

   We thank the Reviewer for these kind words and are happy that he/she appreciates our results. We share his/her opinion that advanced theoretical treatments of biologically relevant models should have their place in PLoS CB.

3. ***Limited range of CV*** *The authors show in their new Fig. 8 – in response to the reviewer's request – and state in the reply that the theory "...predicts the SCC quantitatively up to CVs of about 0.2". This clearly is a rather low value for central brain neurons, e.g. in cortex. However, the qualitative results for the shown cases of higher CVs are still valid. When discussing the limitations of their approach, the authors state clearly that they had to exclude "neurons that are subject to strong stochastic input such as emerging from synaptic background noise in recurrent neural networks..." The high CVs in e.g. cortical neurons is indeed a result of the synaptic input noise. I thus understand that many neurons that do show high CVs empirically are excluded as non-treatable with the present approach.*

   At this point of the Discussion I therefore suggest to add reference to the limitations in CV up to values of 0.2 in a similar manner as the authors did so in response to the reviewer's point 3 (1st revision). The authors may indicate that higher values are typically observed in neuron types that are explicitly excluded here.

   We have added a reference to the limitations of our theory rather early in our manuscript on p. 5

   > Generally and in line with our *weak* noise assumption we discuss cases where the noise intensity is rather small. However, in a later Section we explore the range of validity of our theory in terms of the output variability of the spike train and find quantitative agreement up to $C_V \approx 0.2$. Qualitatively, typical correlation patterns (i.e. SCC as a function of the lag) are well described for an even larger values of $C_V \approx 0.7$.

4. ***Completeness of perturbation expansion approach*** *The major critical point of reviewer 2 in my opinion focuses on the question whether the approach taken here is methodologically appropriate. Reviewer 2 suggests that the perturbation expansion approach is incomplete. He argues that a correct theory requires to consider perturbation in the orthogonal direction. I understood the reviewer's argument; However, I could not resolve whether the authors are correct in their way when stating "our theory is correct in leading order of the perturbation amplitude".*

   My suggestion: Possibly, and more prominently in the Discussion or less prominently in the Methods section, the authors could rephrase and shorten what they are stating in response to the reviewer ("2.

Mathematical correctness"). This statement may then be disputed at any time and by anyone who would be able to proof that the approach taken here is incomplete; or confirm the correctness. That, in my opinion, would be a normal and desired course of scientific progress.

We agree with the Reviewer and would even be happy if our discussion with the second Reviewer would be made public. We have added a passage in the Method section on p.25:

> We stress again that our theory is based on the linear response function of a phase model, namely the phase-response curve, a description that holds true only in leading order of the perturbation amplitude (e.g. σ in the case of purely colored noise). Therefore, in this linear framework, deviations in the phase and in consequence in the interspike-interval caused by perturbations with amplitude σ can meaningfully be described only up to linear order in σ. This also implies that averages of products of intervals depend in leading order on σ^2 . Any extension into the realm of higher-order terms must be based on nonlinear response functions.

5. ***Generality*** *Another aspect raised by the reviewer is that of generality. The authors already made clear restrictions with respect to the applicability of their approach in the above-mentioned Discussion paragraph.*

   Still, I believe the authors could strengthen their manuscript if they demonstrate applicability to a conceptually different type of relevant neuron model, e.g. a conductance-based model, possibly the Wang-Buzsaki model? This would, of course, imply quite a bit of additional work and thus I want to make clear that this is meant as a constructive suggestion rather than a request.

   We thank the Reviewer for the suggestion to consider a different type of neuron model and agree that demonstrating applicability to a conductance-based model does improve the manuscript. We now consider the widely used Traub-Miles model endowed with an slow adaptation current (Ermentrout *Neural Computation*, 1998) in a stochastic version with a mixture of colored and white current noise, see the new section **Traub-Miles model with an M current and both colored and white noise**. Our results show that our theory can indeed be applied to a conductance-based model.

   We have also added a sentence at the end of the Abstract:

   > ~~Finally,~~ We also discuss the range of validity of our weak-noise theory and show that by changing the relative strength of white and colored noise sources, we can change the sign of the correlation coefficient. Finally, we apply our theory to a conductance-based model which demonstrates its broad applicability.

   as well as to the Introduction:

   > ~~Finally,~~ We also discuss the case of a slow population of stochastic ion channels that can be approximated by our model. Finally, we demonstrate that our theory can be applied to a conceptually different model, namely the conductance-based Traub-Miles model with an M current.

Further changes

We have also included a more recent Review on the computational role of adaptation mechanism in Neurons on p. 3

> Adaptation currents include calcium-gated potassium currents, M-Type currents as well as the slow recovery of sodium channels [2] (for the computational role of these and other neural adaptation mechanisms, see [18]).

References

[1] R. Ratnam and M. E. Nelson. "Nonrenewal Statistics of Electrosensory Afferent Spike Trains: Implications for the Detection of Weak Sensory Signals". In: *J. Neurosci.* 20 (2000), p. 6672.

[2] J. Benda and A. V. M. Herz. “A universal model for spike-frequency adaptation”. In: *Neural Comput.* 15 (2003), p. 2523.

[3] M. J. Chacron, B. Lindner, and A. Longtin. “Noise shaping by interval correlations increases information transfer”. In: *Phys. Rev. Lett.* 93 (2004), p. 059904.

[4] B. Lindner, M. J. Chacron, and A. Longtin. “Integrate-and-fire neurons with threshold noise - A tractable model of how interspike interval correlations affect neuronal signal transmission”. In: *Phys. Rev. E.* 72 (2005), p. 021911.

[5] E. M. Izhikevich. *Dynamical Systems in Neuroscience: The Geometry of Excitability and Bursting.* Cambridge, London: The MIT Press, 2007.

[6] Roberto F Galán. “Analytical calculation of the frequency shift in phase oscillators driven by colored noise: Implications for electrical engineering and neuroscience”. In: *Phys. Rev. E* 80.3 (2009), p. 036113.

[7] G Bard Ermentrout and David H Terman. *Mathematical foundations of neuroscience.* Vol. 35. Springer Science & Business Media, 2010.

[8] A. Litwin-Kumar and B. Doiron. “Slow dynamics and high variability in balanced cortical networks with clustered connections”. In: *Nat. Neurosci.* 15 (2012), p. 1498.

[9] T. Schwalger and B. Lindner. “Patterns of interval correlations in neural oscillators with adaptation”. In: *Front. Comp. Neurosci.* 7 (2013), p. 164.

[10] B. Dummer, S. Wieland, and B. Lindner. “Self-consistent determination of the spike-train power spectrum in a neural network with sparse connectivity”. In: *Front. Comp. Neurosci.* 8 (2014), p. 104.

[11] S. Ostojic. “Two types of asynchronous activity in networks of excitatory and inhibitory spiking neurons”. In: *Nat. Neurosci.* 17 (2014), p. 594.

[12] T. Schwalger, F. Droste, and B. Lindner. “Statistical structure of neural spiking under non-Poissonian or other non-white stimulation”. In: *J. Comput. Neurosci.* 39 (2015), p. 29.

[13] S. Wieland et al. “Slow fluctuations in recurrent networks of spiking neurons”. In: *Phys. Rev. E* 92 (2015), 040901(R).

[14] S. Blankenburg and B. Lindner. “The effect of positive interspike interval correlations on neuronal information transmission”. In: *Math. Biosci. Eng.* 13 (2016), p. 461.

[15] B. Lindner. “Mechanisms of Information Filtering in Neural Systems”. In: *IEEE Trans. Mol. Biol. Multi-Scale Commun.* 2 (2016), p. 5.

[16] W. Braun and A. Longtin. “Interspike interval correlations in networks of inhibitory integrate-and-fire neurons”. In: *Phys. Rev. E* 99.3 (2019), p. 032402.

[17] P. Muscinelli, W. Gerstner, and T. Schwalger. “How single neuron properties shape chaotic dynamics and signal transmission in random neural networks”. In: *PLoS Comput. Biol.* 15.6 (2019), e1007122.

[18] J. Benda. “Neural adaptation”. In: *Curr.* 31.3 (2021), R110–R116.
